# Identification of transporters involved in aromatic compounds tolerance through screening of transporter deletion libraries

**DOI:** 10.1111/1751-7915.14460

**Published:** 2024-04-18

**Authors:** Javier Sáez‐Sáez, Lachlan Jake Munro, Iben Møller‐Hansen, Douglas B. Kell, Irina Borodina

**Affiliations:** ^1^ The Novo Nordisk Foundation Center for Biosustainability Technical University of Denmark Kgs. Lyngby Denmark; ^2^ Institute of Systems, Molecular and Integrative Biology University of Liverpool Liverpool UK

## Abstract

Aromatic compounds are used in pharmaceutical, food, textile and other industries. Increased demand has sparked interest in exploring biotechnological approaches for their sustainable production as an alternative to chemical synthesis from petrochemicals or plant extraction. These aromatic products may be toxic to microorganisms, which complicates their production in cell factories. In this study, we analysed the toxicity of multiple aromatic compounds in common production hosts. Next, we screened a subset of toxic aromatics, namely 2‐phenylethanol, 4‐tyrosol, benzyl alcohol, berberine and vanillin, against transporter deletion libraries in *Escherichia coli* and *Saccharomyces cerevisiae*. We identified multiple transporter deletions that modulate the tolerance of the cells towards these compounds. Lastly, we engineered transporters responsible for 2‐phenylethanol tolerance in yeast and showed improved 2‐phenylethanol bioconversion from L‐phenylalanine, with deletions of *YIA6*, *PTR2* or *MCH4* genes improving titre by 8–12% and specific yield by 38–57%. Our findings provide insights into transporters as targets for improving the production of aromatic compounds in microbial cell factories.

## Graphical abstract

Numerous valuable aromatic compounds pose toxicity challenges to microbial cell factories, leading to diminished bioprocess metrics. Membrane transporters play a pivotal role in product tolerance. Leveraging a high‐throughput screening approach on a transporter deletion library, we pinpointed key transporters that modulate product tolerance. Subsequently, we applied these insights to enhance a bioconversion process for 2‐phenylethanol, resulting in significant improvements in bioprocess metrics.

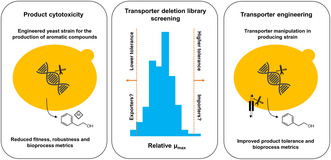

## INTRODUCTION

Aromatic compounds hold significant importance in various aspects of human life, such as food, pharmaceuticals and personal care (Berger, 2007; Liu, 2022; Welsch et al., 2010). As the demand for these compounds rises, the potential of biotechnological manufacturing as an increasingly popular production method has garnered attention, although its practical implementation at an industrial scale remains limited (Averesch & Krömer, 2018; Evolva, 2023a, 2023b; Huccetogullari et al., 2019; Liu et al., 2020). The challenges hindering their production are multifaceted, and one common issue is product cytotoxicity in strain engineering (Kim et al., 2023; Patra, 2012). Many valuable aromatic compounds, often naturally produced by plants as a defence mechanism against biotic stress, tend to display toxicity to microbial cells (Patra, 2012).

To address the issue of product cytotoxicity, tolerance adaptive laboratory evolution (TALE) has been adopted as the preferred method. TALE involves exposing strains to increasing levels of toxic compounds, gradually selecting cells with higher tolerance and ultimately leading to improved cell fitness and robustness (Dragosits & Mattanovich, 2013; Sandberg et al., 2019). However, evolved cells might not be directly suitable as production hosts due to multiple mutations. Some of these mutations may hinder the production of the toxic metabolite, leading to potential issues such as reduced transformation efficiencies, loss of heterologous pathways or decreased product transport. This requires a costly and time‐consuming procedure involving genome re‐sequencing and reverse engineering for the identification and testing of causal mutations in the production strain (Dragosits & Mattanovich, 2013; Sandberg et al., 2019).

In many cases, the genetic basis of tolerance involves mutations in transporter‐encoding genes responsible for exchanging the compound across cellular membranes (Kell, 2021; Lennen et al., 2023; Pereira et al., 2019, 2020; Radi, Munro, et al., 2022; Radi, SalcedoSora, et al., 2022). Beyond improving product tolerance, transporter engineering can offer multiple advantages, including alleviating feedback inhibition, reducing downstream processing costs and preventing the leakage of pathway intermediates, all of which can improve the economics of bioprocesses (Kell, 2018, 2021; Kell et al., 2015; Munro & Kell, 2021; van der Hoek & Borodina, 2020; Zhu et al., 2020).

Despite the numerous benefits of transporter engineering, it remains underutilized in strain engineering cycles due to a lack of knowledge about transporter substrate specificities. Various strategies aside from TALE have been employed to associate substrates with their respective transporters, including toxicity‐based screening of transporter knockout or metagenomic libraries (Acton et al., 2017; Malla et al., 2022; Munro & Kell, 2022; Yang et al., 2022). More recently, genetically encoded biosensors have facilitated the discovery of new transporters for specific compounds by linking intracellular compound concentrations to detectable signals (Genee et al., 2016; Wang et al., 2021). While there has been some investigation into the transport of toxic aromatics, particularly in lignocellulosic biomass conversion and environmental remediation (Mutanda et al., 2022), transporter engineering in the context of microbial production of aromatics remains relatively unexplored. There are successful examples in alkaloid production, which have involved the expression of exporters for final products or importers for pathway intermediates, often in co‐culture systems with two strains (Belew et al., 2020; Dastmalchi et al., 2019; Yamada et al., 2021).

In this, study we performed a preliminary screening on the toxicity of 54 aromatic compounds in *E. coli*, *S. cerevisiae* and *Y. lipolytica*. To identify transporter‐encoding genes that influence product tolerance, *E. coli* and *S. cerevisiae* transporter‐knockout collections were used to screen against a subset of toxic aromatics. The results demonstrated the utility of transporter engineering in improving the strain tolerance against toxic aromatics and how it can help the production of 2‐phenylethanol in *S. cerevisiae*, resulting in enhanced process titre and specific yield.

## RESULTS AND DISCUSSION

### Screening of transporter deletion libraries uncovers transporters that modulate tolerance to aromatics

In recent years, microbial fermentation has garnered attention for sustainably producing aromatic compounds. A literature review in 2019 identified over 50 aromatics produced in cell factories (Huccetogullari et al., 2019). While some studies hint at low‐concentration toxicity for specific compounds, a comprehensive assessment of aromatic compound toxicity in diverse microbial hosts is absent.

To address this gap, we conducted a thorough evaluation of the toxicity of 54 aromatic compounds, considering diverse ring structures, functional groups, commercial relevance and potential for cell factory production (Tables S1–S3). For the toxicity assessment, we selected common production hosts: *Escherichia coli* (strains BL21(DE3) and K‐12 MG1655), *Saccharomyces cerevisiae* (Averesch & Krömer, 2018; Huccetogullari et al., 2019; Liu et al., 2020), and the oleaginous yeast *Yarrowia lipolytica*, known for its excellent potential in aromatics production (Gu et al., 2020; Liu et al., 2022; Sáez‐Sáez et al., 2020). In general, we found no major differences in tolerance among the different microorganisms tested, and compounds that were toxic to only one host were rare occurrences (Figure S1). Notably, *Y. lipolytica* appeared to be equally or more tolerant than *S. cerevisiae*, while *E. coli* strains showed similar tolerance levels, with some specific compounds exhibiting differential effects on the two strains. Comparable strain‐specific variations in *E. coli* have also been reported for the tolerance against compounds including NaCl or H_2_O_2_ (Lennen & Herrgård, 2014). A multi‐omics analysis of multiple *E. coli* strains revealed notable molecular differences, providing a probable explanation for the observed variations in tolerance (Monk et al., 2016).

Identifying toxic aromatic compounds for microbial production hosts prompted the development of a strategy for generating tolerant strains. Utilizing evidence that transporter manipulation affects resistance or sensitivity to toxic compounds, we screened transporter deletion libraries covering a substantial portion of annotated transporter genes (Lennen et al., 2023; Pereira et al., 2019, 2020; Radi, SalcedoSora, et al., 2022). Specifically, we screened 444 *E. coli* and 305 *S. cerevisiae* individual transporter knockout strains, derived from the Keio and YKO collections, respectively (Table S4, Figure S2a,b) (Munro & Kell, 2022; Wang et al., 2021; Yang et al., 2022).

The transporter deletion library was screened against a subset of aromatic compounds from those identified as toxic (Figure S1). Our selection criteria were based on multiple factors, including industrial relevance and reports of production and toxicity in cell factories. The compounds we selected were: 2‐phenylethanol, 4‐tyrosol, benzyl alcohol, berberine and vanillin (Table S2).

**FIGURE 1:**
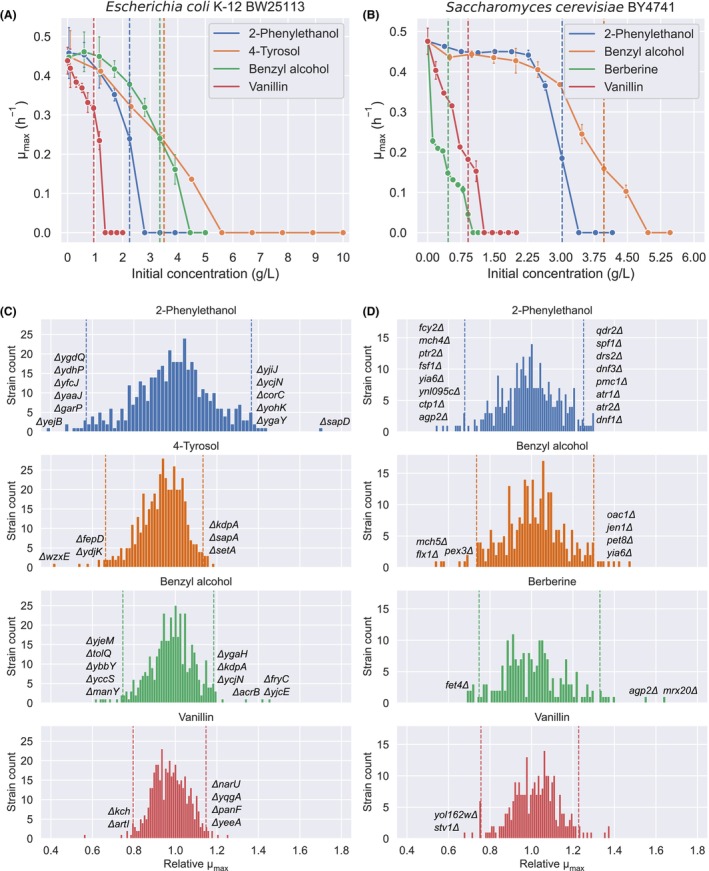
Screening the transporter deletion library for tolerance against toxic aromatic compounds. Panels (A) and (B) display the toxicity of each aromatic compound towards the parent strain of the transporters deletion library, with the dashed lines indicating the chosen concentration for subsequent library screening. *E. coli* concentrations: 2.25 g/L 2‐phenylethanol. 3.5 g/L, 4‐tyrosol, 3.35 g/L benzyl alcohol, 0.95 g/L vanillin. *S. cerevisiae* concentrations: 3.03 g/L 2‐phenylethanol, 3.97 g/L benzyl alcohol, 0.46 g/L berberine, 0.91 g/L vanillin. Panels (C) (*E. coli*) and (D) (*S. cerevisiae*) depict the distribution of the library against each aromatic compound through histograms. The mean relative *μ*_max_ with respect to the wild‐type control is used as the distribution parameter. The relative *μ*_max_ cutoff value, indicated by dashed lines, was used to select the top eight candidates with the highest and lowest product tolerance. Strains from each top 8 with validated effects are identified by labelled histogram bins (Figure 2).

As the genetic background of the parent strains from the Keio and YKO collections differed from those used in the toxicity screening, resulting in potential variations in tolerance, we reassessed the compound toxicity in the new backgrounds (Figure 1A,B). For the library screening, we selected a compound concentration that resulted in ca. halving of the maximum specific growth rate (*μ*_max_), with the aim of identifying transporter deletions that could improve or reduce product tolerance (Figure 1A,B, Table S5). For each strain of the library, we analysed the *μ*_max_ relative to the wild‐type strain in the presence of each toxic compound (Figure 1C,D). In general, we observed a normal library population distribution, with most strains falling within ±2 standard deviations of the mean relative *μ*_max_ value. To identify potential transporters responsible for transporting aromatics, we selected for further validation the top 8 strains for each microorganism and compound with the highest and lowest product tolerance.

Although the library screening method allowed for identifying preliminary transporter deletions affecting product tolerance, it had some limitations that could have affected the *μ*_max_ determinations. The lack of a pre‐culture step and the direct growth of cells from cryostocks, which could have led to differences in inoculum size and state, have been demonstrated to result in significant variations in *μ*_max_ in high‐throughput screenings (Atolia et al., 2020). To address these limitations, in the validation step of the selected candidates, we used the same inocula size of exponentially growing cells for all strains, and we increased the number of replicates to ensure sufficient statistical power.

**FIGURE 2:**
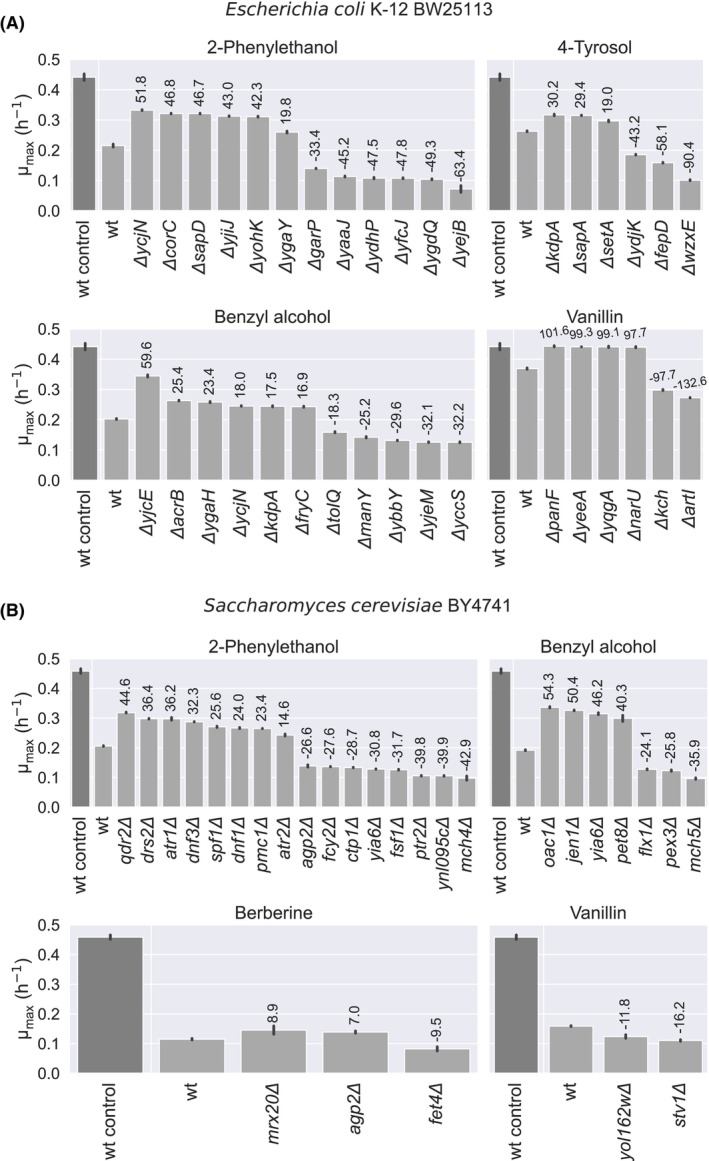
Contribution of transporter deletions to aromatics tolerance in (A) *E. coli* and (B) *S. cerevisiae*. Values on top of the bars show the percentage of recovered tolerance, using as references the wild‐type strain in the presence of the toxic aromatic compounds (wt, 0%) and the wild‐type strain in the absence of aromatics (wt control, 100%). Error bars indicate the standard deviation of at least four biological replicates. All the transporter‐encoding gene deletions, either enhancing or diminishing product tolerance, exhibited a statistically significant difference in *μ*_max_ with respect to the wild‐type strain in the presence of the toxic aromatic compounds (wt, 0%) (*p*‐value <0.001, two‐tailed, unequal variance).

To confirm that the effects observed in the relative *μ*_max_ were not due to a growth defect of the transporter deletion, we evaluated the impact of each candidate transporter deletion in the absence of any aromatic compound (Figure S3a,b). Most transporter deletions did not cause significant differences in *μ*_max_ compared to the wild‐type strain when grown in the control medium. However, marked differences were observed in the strains harbouring deletions of *ygdQ*, *tolQ* and *ybbY* in *E. coli* and deletions in *PTR2*, *YNL095C* and *FLX1* in *S. cerevisiae*, with up to ca. 25% reduction in *μ*_max_. Next, we assessed the effect of the transporter deletions in the presence of toxic aromatics and confirmed several deletions resulting in improved or reduced tolerance for specific compounds (Figure 2A,B and Figures S3, S4a,b, Table S6). Transporter deletions were generally found to restore *μ*_max_ by app. 15–60%.

As expected due to the promiscuity associated with transporters, specific transporter deletions were found to affect the tolerance of more than one aromatic, like *ΔycjN* in *E. coli* or *agp2Δ* in *S. cerevisiae*. Applying a similar rationale, the analysis of extensive datasets can identify multiple strains with single transporter‐encoding gene deletions exhibiting consistent behaviour across various toxic aromatics (Figures S5a,b–S7). This approach has the potential to unveil the function of transporters with unknown functionalities by drawing comparisons with those of known functions.

Some of the transporter‐encoding gene deletions affecting product tolerance were consistent with previously reported data on other compounds with aromatic rings. For instance, the deletion of *garP* sensitized *E. coli* towards 2‐phenylethanol, an effect also observed for melatonin (Yang et al., 2022). In yeast, disruption of *QDR2* improved the tolerance towards 2‐phenylethanol in this work. This gene was previously linked to the transport of other aromatics like betaxanthins (Savitskaya et al., 2019; Wang et al., 2021).

Although it may appear intuitive that transporter genes deleted leading to increased tolerance encode importers and those resulting in enhanced sensitivity encode exporters, numerous studies have demonstrated that single gene deletions can have pleiotropic effects on the expression of multiple other genes (Barrio‐Hernandez et al., 2023; Cooper et al., 2007; Featherstone & Broadie, 2002). Thus, follow‐up validation experiments are necessary to confirm that the observed effect on tolerance results directly from altered transport. Expressing transporters in *Xenopus* oocytes and conducting transport assays would be valuable for validating this mechanism (Miller & Zhou, 2000; Wang et al., 2021). Noteworthy, exporters are far more likely to turn up in toxicity screening assays since if multiple importers exist, the loss of one simply lets others ‘take up the slack’ (Mendes et al., 2020).

Regardless of the underlying mechanism of product tolerance, our method provides an efficient alternative to traditional approaches like TALE, bypassing labour‐intensive tasks and offering a cost‐effective solution for achieving product tolerance in transporter‐encoding gene deletions (Figure 2) (Babel & Krömer, 2020; Lennen et al., 2023). This eliminates the need for resource‐intensive steps such as serial transfers, genome sequencing, mutation identification and reverse engineering (Dragosits & Mattanovich, 2013; Mohamed et al., 2017, 2020; Radi, Munro, et al., 2022; Sandberg et al., 2019).

### Transporter engineering modulates 2‐phenylethanol production in *S. cerevisiae*

**FIGURE 3:**
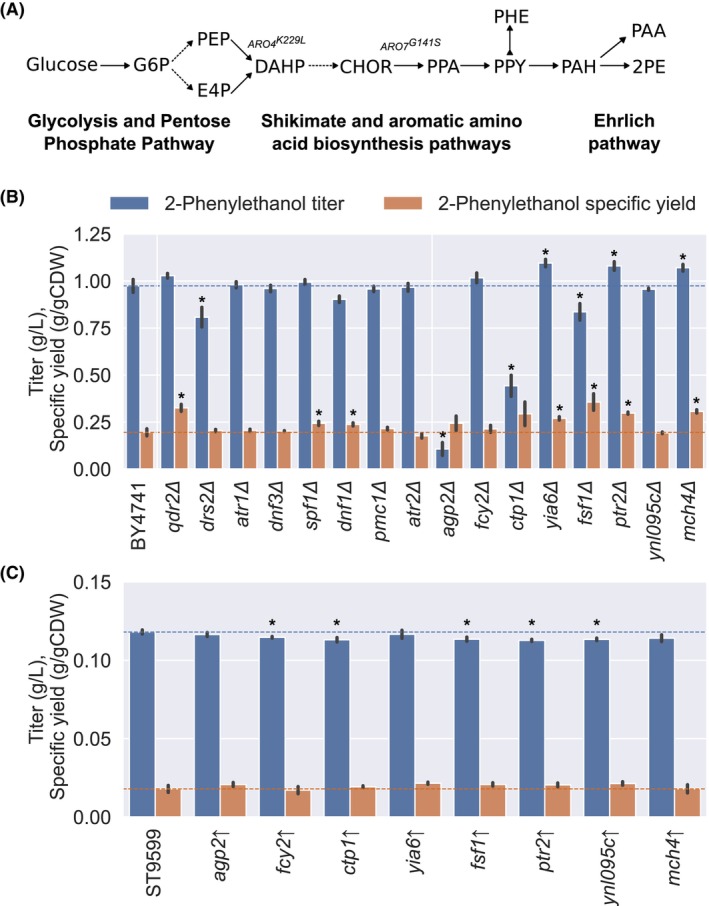
Effect of transporter engineering on 2‐phenylethanol production in *S. cerevisiae*. (A) Metabolic pathways leading to 2‐phenylethanol biosynthesis in *S. cerevisiae*. Dashed lines indicate multiple enzymatic steps. CHOR, chorismate; DAHP, 3‐Deoxy‐D‐arabinoheptulosonate 7‐phosphate; G6P, glucose 6‐phosphate; PEP, phosphoenolpyruvate; erythrose 4‐phosphate; PPA, prephenate; PPY, phenylpyruvate; PHE, L‐phenylalanine; PAH, phenylacetaldehyde; PAA, phenylacetate; 2PE, 2‐phenylethanol. (B) Effect of transporters deletions on 2‐phenylethanol bioconversion. (C) Effect of transporters overexpressions on 2‐phenylethanol bioconversion. L‐phenylalanine was supplemented at a concentration of 5 g/L. Asterisks (*) indicate a *p*‐value <0.05 (two‐sample unequal variance).

2‐Phenylethanol is a naturally occurring metabolite synthesized by *S. cerevisiae* and other yeasts via the Ehrlich pathway, utilizing the aromatic amino acid L‐phenylalanine as a precursor (Dai et al., 2021) (Figure 3A). This compound is interesting for cosmetics and fragrance industries and for fermented alcoholic beverages such as wine (Cordente et al., 2021; Mitri et al., 2022).

Our transporters deletion library identified 16 transporter deletions altering the tolerance of *S. cerevisiae* to exogenously supplemented 2‐phenylethanol (Figure 2B). Subsequently, we investigated whether these changes could also impact its production. Although 2‐phenylethanol can be produced natively through the Ehrlich pathway, the production is typically low in wild‐type strains, and extensive genetic engineering is required to achieve significant titers (Hassing et al., 2019). Therefore, we sought an alternative approach and decided to use whole‐cell bioconversion by supplementing L‐phenylalanine to the medium. In other studies, this approach previously resulted in gram‐per‐litre titers (Cui et al., 2011; Mitri et al., 2022). We tested the 16 deletion strains in the presence and absence of 5 g/L of L‐phenylalanine. Also, with the hypothesis that the eight transporter deletions leading to increased sensitivity could be 2‐phenylethanol exporters, we overexpressed them in a strain with improved flux within the shikimate pathway (strain ST9599: CEN.PK113‐7D + ARO4^K229L^ + ARO7^G141S^) (Luttik et al., 2008).

The deletion of *YIA6*, *PTR2* and *MCH4* led to a significant improvement in titers, with up to a 12.4% increase, as well as specific yield, with an increase of up to 82.2% (Figure 3B). Disrupting *QDR2*, *DNF1* and *SPF1* only improved specific yield. On the other hand, deletions of *DRS2*, *CTP1*, *FSF1* and *AGP2* reduced 2‐phenylethanol titers. Interestingly, strains with these deletions, especially *AGP2* and *CTP1*, exhibited significant growth impairment, an effect that was not observed in the control medium without L‐phenylalanine (Figure S8). Ptr2p and Agp2p stand out due to their roles in peptide and amino acid import and nitrogen metabolism (Aouida et al., 2005, 2013; Becerra‐Rodríguez et al., 2020; Sáenz et al., 2014). Agp2p, in particular, has been speculated to be involved in the uptake of L‐phenylalanine (Schreve & Garrett, 2004), which could explain the impaired growth of the *agp2Δ* strain in excess of extracellular L‐phenylalanine (Figure S8). The overexpression of transporters did not lead to improvements in the measured metrics. In fact, overexpression of *FCY2*, *CTP1*, *FSF1*, *YNL095C* and *MCH4* resulted in lower titers (Figure 3C). The structural similarity between L‐phenylalanine and 2‐phenylethanol may indicate the involvement of certain identified transporters in the transport of both molecules, providing a potential explanation for the somewhat counterintuitive results observed.

The toxicity of 2‐phenylethanol in *S. cerevisiae* and the identification of tolerance mechanisms have been the focus of several recent studies (Xia et al., 2022; Zhu et al., 2021). A missense mutation in *PDR1* was demonstrated to improve titers in a producing strain (Xia et al., 2022). *PDR1* is a transcription factor regulating the expression of multiple genes, and among them, there are ATP‐binding cassette (ABC) transporters like *PDR5*, *PDR15*, *PDR10*, *PDR12*, *PDR11*, *YOR1* and *AUS1*, which confer resistance to several drugs (Buechel & Pinkett, 2020). Interestingly, our library screening did not reveal any of these transporter deletions affecting 2‐phenylethanol tolerance, as probably a change in the expression of all transporters regulated by *PDR1* is required to observe a similar effect.

## CONCLUSIONS

A standard systems biology strategy first identifies genes relevant to the process and then seeks to manipulate them. This study accordingly provides valuable insights into the toxicity of aromatics in common microbial production hosts and identifies specific transporter‐encoding genes involved in product tolerance. Our findings suggest that transporter engineering could be a promising strategy to improve the production of aromatic compounds in microbial cell factories. Further research can explore the potential of the identified transporters as targets for engineering.

## AUTHOR CONTRIBUTIONS

**Javier Sáez‐Sáez:** Conceptualization; investigation; methodology; validation; writing – original draft; writing – review and editing. **Lachlan Jake Munro:** Conceptualization; investigation; methodology; writing – review and editing. **Iben Møller‐Hansen:** Conceptualization; writing – review and editing. **Douglas B. Kell:** Conceptualization; writing – review and editing. **Irina Borodina:** Conceptualization; funding acquisition; project administration; supervision; writing – original draft; writing – review and editing.

## CONFLICT OF INTEREST STATEMENT

The authors declare that they have no conflicts of interest to disclose.

## Supporting information


Data S1:
