# Supplementary material for: Identification of transporters involved in aromatic compounds tolerance through screening of transporter deletion libraries
Source: Microb Biotechnol. 2024 Apr 18;17(4):e14460. doi: 10.1111/1751-7915.14460 (PMC11025615; doi:10.1111/1751-7915.14460)
Supplement: Supplementary file 1 — Data S1: [file MBT2-17-e14460-s001.docx]

**Experimental Procedures**

**Strains and media**

The initial toxicity assays were performed using *Saccharomyces cerevisiae* CEN.PK113-7D, *Yarrowia lipolytica* W29 (NRRL Y-63746), and *Escherichia coli* K-12 MG1655 and BL21(DE3) (Entian and Kötter, 2007; Gaillardin et al., 1973; Bachmann, 1972; Studier and Moffatt, 1986). For tolerance screenings against a selected group of toxic aromatics, isolates harboring single deletions in transporter-encoding genes were obtained from the YKO (*S. cerevisiae* BY4741) and Keio (*E. coli* K-12 BW25113) deletion collections (Baba et al., 2006; Winzeler et al., 1999; Giaever et al., 2002). *S. cerevisiae* CEN.PK113-7D strain background was used for 2-phenylethanol overproduction and for testing the selected transporter candidates. All the strain genotypes are described in Table S7.

Yeast strains were cultivated in mineral medium containing (per L^-1^) 7.5 g (NH_4_)_2_SO_4_, 14.4 g KH_2_PO_4_, 0.5 g MgSO_4_·7H_2_O, 20 g D-glucose, 2 mL trace metals solution, and 1 mL vitamins. The trace metals and vitamins solution were prepared as previously described (Jensen et al., 2014). The pH of the medium was adjusted to pH 6.0 with KOH. Auxotrophies of *S. cerevisiae* BY4741 derived strains were complemented by supplementing the medium with 380 mg/L leucine, 76 mg/L histidine, 76 mg/L methionine, and 76 mg/L uracil. In the production assays of 2-phenylethanol, 5 g/L of L-phenylalanine was added to the medium for bioconversion.

*E. coli* was grown in M9extra medium containing (per L^-1^) 12.8 g Na_2_HPO_4_·7H_2_O, 3 g KH_2_PO_4_, 0.5 g NaCl, 1 g NH_4_Cl, 0.24 g MgSO_4_, 0.011 g CaCl_2_, 20 g D-glucose, 1 mL FeCl_3_ solution (50 mM FeCl_3_·6H_2_O in 100 mM citric acid monohydrate), and 500 µL of a trace elements solution, as previously described (Falkenberg et al., 2021). The pH of the medium was adjusted to pH 6.8 with NaOH.

Aromatic compounds in toxicity and tolerance screenings were added to the media and solubilized. The pH was then adjusted and the media were filter-sterilized. All the aromatic compounds were purchased from Sigma-Aldrich.

**Strain construction**

To construct integration plasmids for the overexpression of candidate transporter genes, we utilized the EasyClone-MarkerFree cloning system (Jessop‐Fabre et al., 2016). Integration fragments and gRNA plasmids for CRISPR/Cas9-mediated genomic integration were constructed according to the protocol described by Jessop-Fabre et al. (Jessop‐Fabre et al., 2016). BioBricks were amplified with Phusion U Hot Start DNA Polymerase (Thermo Fisher Scientific) and USER-compatible primers, and then subjected to USER reactions based on the standard protocol from New England Biolabs. The resulting products were transformed into competent *E. coli* DH5α cells to assemble and propagate the plasmids, which were then purified using the NucleoSpin plasmid miniprep kit (Macherey Nagel) and sequenced by Eurofins Scientific to confirm the correct plasmid assembly. FastDigest NotI restriction enzyme (Thermo Fisher Scientific) was used to linearize the integration fragments, which were then transformed into *S. cerevisiae* strains expressing Cas9, along with the corresponding gRNA plasmids, using the LiAc/ssDNA/PEG method (Gietz and Schiestl, 2007). Transformations were recovered in YPD+G418 media at 30°C with 250 rpm shaking before plating on YPD supplemented with G418 and nourseothricin. We confirmed the integration of the vectors into the correct sites on the genome by performing colony PCR with RedTaq DNA polymerase (VWR Life Science). We provide a comprehensive list of all strains, plasmids, BioBricks, and primers used and constructed in this study in the Supporting Information (Tables S7–S10).

**Cultivation procedure and conditions**

In the toxicity experiments, 0.5 mL of medium in a 13 mL pre-culture tube was inoculated with wild-type strains, stored as cryostocks, using a 10 µL inoculation loop. The tubes were incubated at 30 (yeast) or 37 °C (*E. coli*) and 250 rpm for 20 (*E. coli*) or 24 hours (yeast). Next, 400 µL of broth was transferred to a 250 mL shake flask containing 10 mL of medium and incubated under the same conditions for 6-8 (*E. coli*) or 10-12 hours (yeast) until having exponentially growing cells. These cultures were then used as inoculum (<5 µL, initial OD_600_ ≈ 0.1) for 96-well plates with transparent bottom (CR1496dg, EnzyScreen BV) containing 300 µL of medium per well. Plates were covered with a sandwich cover with pins (CR1396b, EnzyScreen BV).

To screen the transporter deletion library, 500 µL of medium in 96-deep well plates was inoculated with the library using a 96-pin replicator (CR1000, EnzyScreen BV). The plates were covered with an air penetrable lid (CR1296, EnzyScreen BV) and then incubated at 30 °C (yeast) or 37 °C (*E. coli*) and 300 rpm overnight. The following day, the resulting plates were mixed with 500 µL of glycerol. A 3 µL droplet of the broth and glycerol mixture was dispensed into the bottom of each well in 96-well plates with transparent bottom (CR1496dg, EnzyScreen BV). The loaded plates containing the propagated library were stored at −20 °C for up to 3 weeks, or at −80 °C for longer storage. To initiate the library screening, 297 µL of media was dispensed into the inoculated plates, which prior to incubation were covered with a sandwich cover with pins (CR1396b, EnzyScreen BV). Glycerol mixing of pre-cultures, inoculation and media loading were performed using an Opentron OT-2 liquid handler, as previously described (Munro and Kell, 2022). Scripts for operating the OT-2 have been previously made available at <https://github.com/ljm176/TransporterScreening>.

For the validation of transporter candidates and in the 2-phenylethanol production assays, 96 deep-well plates containing 0.5 mL of medium were inoculated with the strains. The plates were covered with an air penetrable lid (CR1296, EnzyScreen BV) and incubated at 30 (*S. cerevisiae*) or 37 °C (*E. coli*) and 300 rpm for 18 (*E. coli*) or 20 hours (yeast). The following day, OD_600_ of the pre-cultures was measured and a volume corresponding to an initial OD_600_ of 0.1 was transferred to 96-well plates with a transparent bottom (CR1496dg, EnzyScreen BV), containing 300 µL of medium.

In all experiments, plates were incubated using the Growth Profiler 960 (EnzyScreen BV) at 30 °C/250 rpm for yeast and 37 °C/225 rpm for *E. coli*, following the manufacturer’s recommended shaking settings. Images were captured at 15-20 minute intervals for *E. coli* and 20-30 minute intervals for yeast, with a shutter time of 5 ms. Cultivation times were at least 40 hours for *E. coli* and 60 hours for yeast to ensure adequate growth for the estimation of µ_max_. Cultivations were performed in duplicate, with the exception of the validation of transporter-deletion candidates (n = 4) and the assessment of the effect of transporter engineering on 2-phenylethanol production (n = 3).

**Microbial growth data analysis**

Growth Profiler 960 pictures were processed using the manufacturer’s software GP960Viewer to obtain G-values from the central pixels of each well. G-values were then converted to OD_600_ equivalents using calibration curves previously generated. Growth rates were determined from the growth data using custom Python scripts that calculate the slope of linear regression fits between ln-transformed OD_600_ and time. A minimum OD_600_ of 0.125 in *E. coli* and 0.3 in yeast was required to initiate growth rate calculations. A sliding window of 12 time points (corresponding to 180-240 minutes in *E. coli* and 240-360 minutes in yeast) was used to determine growth rates across the growth curves. The maximum specific growth rate (µ_max_) was defined as the maximum slope within the growth profile having a correlation coefficient R^2^ greater than 0.98. Half maximal inhibitory concentrations (IC_50_) were determined using GraphPad Prism 9 and a variable slope four-parameter model. During the screening of the transporter deletion library, any strains that were unable to grow in a control medium without the presence of any toxic aromatic compounds were excluded from further analysis. Additionally, any strains that displayed a coefficient of variation greater than 0.3 for their relative µ_max_ in the presence of aromatics compounds (µ_max_ strain/µ_max_ wild-type) were also excluded from the analysis. The complete set of raw and analyzed data has been made accessible in the supplementary files.

**Quantification of 2-phenylethanol using high-pressure liquid chromatography**

To quantify 2-phenylethanol, the supernatant of the yeast cultivation samples was used after centrifugation at 17,000 g for 5 min. The quantification was performed using a Dionex UltiMate 3000 high-pressure liquid chromatography (HPLC) system equipped with a DAD-3000 UV/Vis detector (Dionex). The stationary phase was a Discovery HS F5 150 mm × 2.1 mm column with a particle size of 3 μm, while the mobile phase was a binary system composed of 10 mM ammonium formate at pH 3.0, adjusted by formic acid (solvent A), and acetonitrile (solvent B). The flow rate was set to 0.7 mL/min, and 5 μL of the sample was injected for quantification. The solvent composition was initially set to A = 95.0% and B = 5.0%, which was maintained until 0.5 min. Thereafter, the solvent composition was changed following a linear gradient until A = 40.0% and B = 60.0% at 7.0 min, which was kept constant for 2.5 min (7.0–9.5 min). The solvent composition was then returned linearly to the initial conditions (A = 95.0%, B = 5.0%) at 9.6 min and remained unchanged until the end of the run (9.6-12 min).

2-Phenylethanol was detected at a retention time of 4.8 min, and the absorbance at 214 nm was used for quantification. Peaks corresponding to the target compounds were identified by comparison to prepared standards (Sigma-Aldrich). Peak areas were used for compound quantification using the external standard calibration method. Analysis of HPLC results was performed using the software Chromeleon 7 (Thermo Fisher Scientific).

**Table S1. List of 54 compounds evaluated for toxicity**. Classes of compounds: A: Phenyl/Benzyl alcohols, amines, aldehydes or other hydrocarbons; B: Phenyl/Benzyl acids or amino acids; C: Indoles; D: Complex secondary metabolites; E: Dicarboxylic acid.

| **Compound** | **Class** | **Chemical structure** |
| --- | --- | --- |
| 2-Phenylacetaldehyde | A |  |
| 2-Phenylethanol | A |  |
| 2-Phenylethylamine | A |  |
| 4-Aminobenzoic acid | B |  |
| 4-Coumaric acid | B |  |
| 4-Tyrosol | A |  |
| 4-Vinylphenol | A |  |
| 5-Hydroxytryptophan | C |  |
| Acetylsalicylic acid | B |  |
| Aspartame | B |  |
| Atenolol | A |  |
| Benzyl alcohol | A |  |
| Berberine | D |  |
| Caffeic acid | B |  |
| Catechin | D |  |
| Catechol | A |  |
| Colchicine | D |  |
| Dopamine | A |  |
| Ferulic acid | B |  |
| Gallic acid | B |  |
| Gastrodin | A |  |
| Hydrocinnamyl alcohol | A |  |
| Isoeugenol | A |  |
| Kaempferol | D |  |
| L-DOPA | B |  |
| Mandelic acid | B |  |
| Melatonin | C |  |
| Methyleugenol | A |  |
| Myricetin | D |  |
| Naringenin | D |  |
| Neotame | A |  |
| Novobiocin | D |  |
| Papaverine | D |  |
| Phenylalanine | B |  |
| Phloretin | D |  |
| Protocatechuic acid | B |  |
| Quercetin | D |  |
| Quercetin 3-glucoside | D |  |
| Quinine | D |  |
| Resveratrol | D |  |
| Rosmarinic acid | D |  |
| Rutin | D |  |
| Salicylic acid | B |  |
| Serotonin | C |  |
| Shikimic acid | B |  |
| Styrene | A |  |
| Tryptophan | C |  |
| Tubocurarine | D |  |
| Tyrosine | B |  |
| Vanillic acid | B |  |
| Vanillin | A |  |
| *cis,cis*-Muconic acid | E |  |
| *trans*-Anethole | A |  |
| *trans*-Cinnamic acid | B |  |

**Table S2. Applications and uses of the 54 compounds evaluated for toxicity**, including microbial production examples, when available. n.a.: not available.

| **Compound** | **Applications and uses** | **Example of microbial production** |
| --- | --- | --- |
| 2-Phenylacetaldehyde | Cosmetics ingredient; floral odor | (Wang et al., 2023) |
| 2-Phenylethanol | Cosmetics ingredient; rose-like odor | (Wang et al., 2019) |
| 2-Phenylethylamine | Central nervous system stimulant | (Hamana and Niitsu, 1999) |
| 4-Aminobenzoic acid | Building block for drugs and pesticides | (Averesch et al., 2016) |
| 4-Coumaric acid | Food ingredient (antioxidant) | (Rodriguez et al., 2015) |
| 4-Tyrosol | Food ingredient (antioxidant) | (Xu et al., 2020) |
| 4-Vinylphenol | Food ingredient (aroma) | (Salgado et al., 2014) |
| 5-Hydroxytryptophan | Antidepressant, appetite suppressant, and sleep aid | (Wang et al., 2018) |
| Acetylsalicylic acid | Anti-inflammatory drug | n.a. |
| Aspartame | Artificial sweetener | n.a. |
| Atenolol | Anti-hypertensive drug | n.a. |
| Benzyl alcohol | General solvent, bacteriostatic preservative for drugs, drug for the treatment of head lice | (Pugh et al., 2015) |
| Berberine | Natural yellow dye, supplement with blood-sugar-lowering properties | (Han and Li, 2023) |
| Caffeic acid | Food supplement used to boost the performance of athletes | (Zhou et al., 2021) |
| Catechin | Food supplement with antioxidant and antimicrobial properties | (Zhao et al., 2015) |
| Catechol | Building block for pesticides, perfumes and drugs | (Song et al., 2022) |
| Colchicine | Drug to treat gout and Behçet's disease | n.a. |
| Dopamine | Neuromodulatory substance | (Trenchard et al., 2015) |
| Ferulic acid | Cosmetics ingredient with antioxidant properties | (Lv et al., 2021) |
| Gallic acid | Tanning, ink dyes, and the manufacture of paper | (Aguilar-Zárate et al., 2015) |
| Gastrodin | Dietary supplement, drug to treat headache and migraine | (Yin et al., 2020) |
| Hydrocinnamyl alcohol | Flavoring agent | (Liu et al., 2021) |
| Isoeugenol | Food additive and cosmetics ingredient | (Wang et al., 2021) |
| Kaempferol | Dietary supplement with multiple health-promoting properties | (Lyu et al., 2019) |
| L-DOPA | Neurotransmitters precursor, used in the treatment of Parkinson’s disease | (Fordjour et al., 2019) |
| Mandelic acid | Cosmetics ingredient | (Sun et al., 2011) |
| Melatonin | Dietary supplement used in the treatment of sleep disorders | (Zhang et al., 2021) |
| Methyleugenol | Food additive and cosmetics ingredient | n.a. |
| Myricetin | Dietary supplement with multiple health-promoting properties | (Leonard et al., 2006) |
| Naringenin | Food supplement with multiple health-promoting properties | (Wei et al., 2020) |
| Neotame | Artificial sweetener | n.a. |
| Novobiocin | Antibiotic | (Steffensky et al., 2000) |
| Papaverine | Drug for the treatment of visceral spasms and vasospasms | (Jamil et al., 2022) |
| Phenylalanine | Amino acid used for the treatment of vitiligo and other disorders | (Liu et al., 2019) |
| Phloretin | Dietary supplement with multiple health-promoting properties | (X. Liu et al., 2022) |
| Protocatechuic acid | Pharmacological applications as antioxidant, anti-inflammatory, and neuroprotective | (Li and Ye, 2021) |
| Quercetin | Dietary supplement with multiple health-promoting properties | (Rodriguez et al., 2017) |
| Quercetin 3-glucoside | Dietary supplement with multiple health-promoting properties | (De Bruyn et al., 2015) |
| Quinine | Drug used to treat malaria and babesiosis | n.a. |
| Resveratrol | Dietary supplement with multiple health-promoting properties | (M. Liu et al., 2022) |
| Rosmarinic acid | Dietary supplement with multiple health-promoting properties | (Babaei et al., 2020) |
| Rutin | Dietary supplement with multiple health-promoting properties | n.a. |
| Salicylic acid | Cosmetics ingredient for the treatment of many skin disorders | (Ahmadi et al., 2016) |
| Serotonin | Multifaceted hormone | (Mora-Villalobos and Zeng, 2018) |
| Shikimic acid | Precursor of antiviral drugs | (Martínez et al., 2015) |
| Styrene | Platform chemical for the manufacturing of latex, synthetic rubber, polystyrene resins, and other plastics | (Lee et al., 2019) |
| Tryptophan | Amino acid used in the treatment of sleep disorders | (Niu et al., 2019) |
| Tubocurarine | Adjunct for clinical anesthesia | n.a. |
| Tyrosine | Amino acid sold as dietary supplement | (Gold et al., 2015) |
| Vanillic acid | Flavoring agent with multiple biological activities | (Weiland et al., 2023) |
| Vanillin | Food additive and cosmetics ingredient | (Brochado et al., 2010) |
| *cis,cis*-Muconic acid | Platform chemical for manufacturing of plastics, resins, and pharmaceuticals | (Wang et al., 2022) |
| *trans*-Anethole | Flavoring agent and sweetener | n.a. |
| *trans*-Cinnamic acid | Food additive and cosmetics ingredient | (Vargas-Tah and Gosset, 2015) |

**Table S3. Concentration range tested for each compound.**

| **Compound** | **C_1_ (g/L)** | **C_2_ (g/L)** | **C_3_ (g/L)** | **C_4_ (g/L)** | **C_5_ (g/L)** |
| --- | --- | --- | --- | --- | --- |
| 2-Phenylacetaldehyde | 2 | 1 | 0.5 | 0.25 | 0.125 |
| 2-Phenylethanol | 5 | 2.5 | 1.25 | 0.625 | 0.3125 |
| 2-Phenylethylamine | 2 | 1 | 0.5 | 0.25 | 0.125 |
| 4-Aminobenzoic acid | 5 | 2.5 | 1.25 | 0.625 | 0.3125 |
| 4-Coumaric acid | 0.8 | 0.4 | 0.2 | 0.1 | 0.05 |
| 4-Tyrosol | 10 | 5 | 2.5 | 1.25 | 0.625 |
| 4-Vinylphenol | 2 | 1 | 0.5 | 0.25 | 0.125 |
| 5-Hydroxytryptophan | 3 | 1.5 | 0.75 | 0.375 | 0.1875 |
| Acetylsalicylic acid | 2.5 | 1.25 | 0.625 | 0.3125 | 0.15625 |
| Aspartame | 5 | 2.5 | 1.25 | 0.625 | 0.3125 |
| Atenolol | 0.2 | 0.1 | 0.05 | 0.025 | 0.0125 |
| Benzyl alcohol | 5 | 2.5 | 1.25 | 0.625 | 0.3125 |
| Berberine | 1.5 | 0.75 | 0.375 | 0.1875 | 0.09375 |
| Caffeic acid | 5 | 2.5 | 1.25 | 0.625 | 0.3125 |
| Catechin | 0.4 | 0.2 | 0.1 | 0.05 | 0.025 |
| Catechol | 1 | 0.5 | 0.25 | 0.125 | 0.0625 |
| Colchicine | 5 | 2.5 | 1.25 | 0.625 | 0.3125 |
| Dopamine | 4 | 2 | 1 | 0.5 | 0.25 |
| Ferulic acid | 0.5 | 0.25 | 0.125 | 0.0625 | 0.03125 |
| Gallic acid | 10 | 5 | 2.5 | 1.25 | 0.625 |
| Gastrodin | 5 | 2.5 | 1.25 | 0.625 | 0.3125 |
| Hydrocinnamyl alcohol | 5 | 2.5 | 1.25 | 0.625 | 0.3125 |
| Isoeugenol | 0.5 | 0.25 | 0.125 | 0.0625 | 0.03125 |
| Kaempferol | 0.05 | 0.025 | 0.0125 | 0.00625 | 0.003125 |
| L-DOPA | 3 | 1.5 | 0.75 | 0.375 | 0.1875 |
| Mandelic acid | 10 | 5 | 2.5 | 1.25 | 0.625 |
| Melatonin | 0.05 | 0.025 | 0.0125 | 0.00625 | 0.003125 |
| Methyleugenol | 0.4 | 0.2 | 0.1 | 0.05 | 0.025 |
| Myricetin | 0.05 | 0.025 | 0.0125 | 0.00625 | 0.003125 |
| Naringenin | 0.1 | 0.05 | 0.025 | 0.0125 | 0.00625 |
| Neotame | 5 | 2.5 | 1.25 | 0.625 | 0.3125 |
| Novobiocin | 0.5 | 0.25 | 0.125 | 0.0625 | 0.03125 |
| Papaverine | 0.5 | 0.25 | 0.125 | 0.0625 | 0.031 |
| Phenylalanine | 10 | 5 | 2.5 | 1.25 | 0.625 |
| Phloretin | 0.1 | 0.05 | 0.025 | 0.0125 | 0.00625 |
| Protocatechuic acid | 5 | 2.5 | 1.25 | 0.625 | 0.3125 |
| Quercetin | 0.05 | 0.025 | 0.0125 | 0.00625 | 0.003125 |
| Quercetin 3-glucoside | 0.2 | 0.1 | 0.05 | 0.025 | 0.0125 |
| Quinine | 0.3 | 0.15 | 0.075 | 0.0375 | 0.01875 |
| Resveratrol | 0.05 | 0.025 | 0.0125 | 0.00625 | 0.003125 |
| Rosmarinic acid | 0.1 | 0.05 | 0.025 | 0.0125 | 0.00625 |
| Rutin | 0.1 | 0.05 | 0.025 | 0.0125 | 0.00625 |
| Salicylic acid | 2.5 | 1.25 | 0.625 | 0.3125 | 0.15625 |
| Serotonin | 5 | 2.5 | 1.25 | 0.625 | 0.3125 |
| Shikimic acid | 5 | 2.5 | 1.25 | 0.625 | 0.3125 |
| Styrene | 0.2 | 0.1 | 0.05 | 0.025 | 0.0125 |
| Tryptophan | 10 | 5 | 2.5 | 1.25 | 0.625 |
| Tubocurarine | 5 | 2.5 | 1.25 | 0.625 | 0.3125 |
| Tyrosine | 0.4 | 0.2 | 0.1 | 0.05 | 0.025 |
| Vanillic acid | 1.25 | 0.625 | 0.3125 | 0.15625 | 0.078125 |
| Vanillin | 3 | 1.5 | 0.75 | 0.375 | 0.1875 |
| *cis,cis*-Muconic acid | 5 | 2.5 | 1.25 | 0.625 | 0.3125 |
| *trans*-Anethole | 0.1 | 0.05 | 0.025 | 0.0125 | 0.00625 |
| *trans*-Cinnamic acid | 0.4 | 0.2 | 0.1 | 0.05 | 0.025 |

**Table S4. List of transporter-encoding genes included in the deletion library.**

| **Microorganism** | **Individual transporter deletions in the library** |
| --- | --- |
| *Escherichia coli* K-12 BW25113 | *aaeA, acrB, acrD, acrF, actP, adeP, adeQ, adiC, agaC, agaV, alsC, alx, ampG, amtB, ansP, aqpZ, araE, araH, araJ, argO, argT, arnE, arnF, aroP, arsB, artI, artJ, artQ, ascF, atoE, atoS, atpB, atpI, barA, bcr, betT, bglF, brnQ, btuC, cadB, caiT, ccmC, chaA, chbC, citT, clcA, clcB, cmtA, cmtB, codB, copA, corA, corC, crr, cusA, cvrA, cycA, cydD, cynX, cysW, dauA, dctA, dcuA, dcuB, dcuC, dcuD, ddpB, ddpC, ddpD, ddpF, dgoT, dhaM, dinF, dlsT, dppB, dsdX, dtpA, dtpB, dtpC, dtpD, eamA, eamB, emrB, emrD, emrY, entS, ettA, eutH, exbB, exbD, exuT, feoB, fepD, fetA, fetB, fieF, focA, focB, frlA, fruA, frvA, frvB, frwB, frwC, frwD, fryA, fryB, fryC, fsr, fucP, gabP, gadC, galP, garP, gdx, ghxP, ghxQ, glnP, glpF, glpT, gltK, gltP, gltS, glvB, glvC, gntP, gntT, gntU, gsiC, guaB, gudP, hcaT, hisM, hofC, hsrA, idnT, kch, kdgT, kdpA, kdpD, kefB, kefC, kgtP, kup, lacY, leuE, livH, livJ, livK, lldP, lplT, lptB, lsrA, lsrC, lysP, macB, malF, malX, manY, mdfA, mdlA, mdlB, mdtB, mdtD, mdtF, mdtG, mdtH, mdtI, mdtJ, mdtK, mdtL, mdtN, mdtO, melB, mepM, metI, mglC, mgtA, mhpT, mlaC, mlaE, mltF, mngA, modB, modF, mscK, mscL, mscM, mscS, mtlA, mtr, murP, nagE, nanT, narK, narU, nepI, nhaB, nikC, nimT, nirC, nlpA, nupG, nupX, oppB, panF, perM, pheP, phnD, phnK, phnL, phoR, pitA, plaP, potB, potE, potH, proP, proW, proY, pstC, psuT, ptsG, ptsI, ptsN, ptsP, putP, puuP, rarD, rbbA, rbsC, rcnA, rcnB, rhaT, rhmT, rhtA, rhtB, rhtC, rutG, sapA, sapB, sapC, sapD, sapF, sbp, sdaC, setA, setB, setC, sgcA, sgcB, sgcC, shiA, sotB, soxR, srlA, sstT, ssuC, sufB, tatC, tdcC, tehA, thiP, tnaB, tolQ, tolR, torT, tqsA, treB, trkH, tsgA, ttdT, tyrP, uacT, ugpA, uhpC, uhpT, uidB, ulaA, uraA, uup, wzxE, xanP, xanQ, xapB, xylE, xylH, yaaJ, yaaU, yadG, yadH, yadI, yadS, yahN, yajR, ybaE, ybaL, ybaT, ybbA, ybbP, ybbW, ybbY, ybhF, ybhG, ybhI, ybhN, ybhR, ybhS, ybiO, ybiR, ybiT, ybjJ, ybjL, ycaD, ycaM, yccA, yccS, ycfT, ycjN, ycjO, ycjP, ycjV, ydcO, ydcS, ydcT, ydcU, ydcV, ydcZ, yddA, yddB, yddG, ydeE, ydfJ, ydgI, ydhC, ydhJ, ydhK, ydhP, ydiK, ydiM, ydiN, ydjE, ydjK, ydjN, ydjX, yeaV, yebQ, yedA, yeeA, yeeE, yeeO, yegH, yegT, yehW, yehY, yejB, yejE, yfbS, yfcC, yfcJ, yfdC, yfdV, yfeH, yfeO, yfjD, ygaH, ygaY, ygaZ, ygbN, ygcS, ygdQ, yggR, yggT, yghD, yghE, yghF, ygiS, ygjI, yhbE, yhdP, yhdW, yhdX, yhdY, yhdZ, yheS, yhfK, yhgE, yhhJ, yhhS, yhhT, yhiD, yhjE, yhjV, yhjX, yiaM, yiaN, yiaV, yibH, yicG, yicJ, yicL, yidE, yidK, yifK, yihN, yihO, yihP, yijE, yjbB, yjcE, yjeH, yjeM, yjfF, yjhB, yjhF, yjiJ, yjjP, ykgG, yneE, ynfA, ynfM, ynjB, ynjC, ynjD, yoaE, yohK, yojI, yphD, yphE, yphF, ypjA, yqcE, yqeG, yqgA, yraQ, yrbG, ytfF, ytfL, ytfT, zitB, zntA, zntB, znuB, zupT* |
| *Saccharomyces cerevisiae* BY4741 | *YAL022C, YAL026C, YAL053W, YAL067C, YBL042C, YBL089W, YBL099W, YBL102W, YBR008C, YBR021W, YBR043C, YBR068C, YBR069C, YBR085W, YBR104W, YBR132C, YBR171W, YBR180W, YBR187W, YBR207W, YBR219C, YBR220C, YBR235W, YBR241C, YBR287W, YBR291C, YBR293W, YBR294W, YBR295W, YBR296C, YBR298C, YCL002C, YCL025C, YCL038C, YCL069W, YCR011C, YCR023C, YCR028C, YCR037C, YCR075C, YCR098C, YDL054C, YDL100C, YDL119C, YDL128W, YDL138W, YDL149W, YDL194W, YDL199C, YDL206W, YDL210W, YDL231C, YDR011W, YDR046C, YDR061W, YDR093W, YDR107C, YDR119W, YDR135C, YDR178W, YDR205W, YDR270W, YDR298C, YDR329C, YDR338C, YDR345C, YDR352W, YDR406W, YDR438W, YDR456W, YDR470C, YDR497C, YDR508C, YDR536W, YEL004W, YEL006W, YEL031W, YEL063C, YEL065W, YER019C-A, YER039C, YER053C, YER056C, YER060W, YER113C, YER119C, YER145C, YER154W, YER166W, YER185W, YFL011W, YFL040W, YFL050C, YFL054C, YFL055W, YFR045W, YGL006W, YGL077C, YGL084C, YGL114W, YGL140C, YGL186C, YGR033C, YGR055W, YGR062C, YGR096W, YGR121C, YGR131W, YGR138C, YGR181W, YGR213C, YGR217W, YGR224W, YGR257C, YGR260W, YGR281W, YGR289C, YHL008C, YHL016C, YHL035C, YHL036W, YHL040C, YHL047C, YHR032W, YHR048W, YHR050W, YHR092C, YHR094C, YHR096C, YIL006W, YIL013C, YIL023C, YIL088C, YIL120W, YIL121W, YIL134W, YIL166C, YIR028W, YJL059W, YJL062W, YJL093C, YJL107C, YJL108C, YJL129C, YJL133W, YJL163C, YJL193W, YJL198W, YJL214W, YJR001W, YJR077C, YJR095W, YJR106W, YJR121W, YJR124C, YJR135W-A, YJR152W, YKL050C, YKL120W, YKL146W, YKL174C, YKL175W, YKL188C, YKL217W, YKL221W, YKR039W, YKR050W, YKR052C, YKR093W, YKR103W, YKR104W, YKR105C, YKR106W, YLL015W, YLL028W, YLL043W, YLL048C, YLL052C, YLL053C, YLL055W, YLL061W, YLR004C, YLR034C, YLR046C, YLR081W, YLR083C, YLR092W, YLR130C, YLR138W, YLR152C, YLR188W, YLR220W, YLR237W, YLR292C, YLR295C, YLR348C, YML018C, YML038C, YML066C, YML081C-A, YML116W, YML123C, YMR011W, YMR034C, YMR054W, YMR056C, YMR088C, YMR155W, YMR162C, YMR166C, YMR177W, YMR221C, YMR241W, YMR243C, YMR253c, YMR279C, YMR319C, YNL003C, YNL065W, YNL070W, YNL083W, YNL095C, YNL101W, YNL121C, YNL125C, YNL142W, YNL268W, YNL270C, YNL275W, YNL291C, YNL318C, YNL321W, YNR013C, YNR039C, YNR055C, YNR056C, YNR062C, YNR070W, YNR072W, YOL020W, YOL060C, YOL075C, YOL077W-A, YOL092W, YOL103W, YOL119C, YOL122C, YOL137W, YOL158C, YOL162W, YOL163W, YOR011W, YOR045W, YOR049C, YOR071C, YOR079C, YOR087W, YOR092W, YOR100C, YOR130C, YOR153W, YOR161C, YOR192C, YOR222W, YOR270C, YOR271C, YOR273C, YOR291W, YOR306C, YOR307C, YOR316C, YOR328W, YOR332W, YOR334W, YOR348C, YOR378W, YPL006W, YPL036W, YPL058C, YPL060W, YPL078C, YPL092W, YPL134C, YPL147W, YPL189W, YPL224C, YPL244C, YPL264C, YPL265W, YPL270W, YPL271W, YPL274W, YPR003C, YPR011C, YPR021C, YPR036W, YPR058W, YPR128C, YPR138C, YPR149W, YPR156C, YPR192W, YPR194C, YPR198W, YPR201W* |

**Table S5. Compound concentrations used in the library screening and subsequent validation experiments.** Values reported in g/L.

| **Compound** | ***E. coli*** | ***S. cerevisiae*** |
| --- | --- | --- |
| 2-Phenylethanol | 2.25 | 3.03 |
| 4-Tyrosol | 3.5 | n.a. |
| Benzyl alcohol | 3.35 | 3.97 |
| Berberine | n.a. | 0.46 |
| Vanillin | 0.95 | 0.91 |

**Table S6. List of transporter deletions validated to improve or reduce product tolerance.** Host: *Ec*: *E. coli*, *Sc*: *S. cerevisiae*; Arom: Toxic aromatic compound; 2PE: 2-Phenylethanol, 4Tyr: 4-Tyrosol; BenzOH: Benzyl alcohol; Van: Vanillin; Berb: Berberine; Tol: Tolerance; ↑: tolerance improved; ↓: tolerance reduced. Substrate, familiy name, transporter class and TC number have been retrieved from transportDB 2.0 (Elbourne et al., 2017).

| **Host** | **Name** | **Arom** | **Tol** | **Substrate** | **Family name** | **Transp. Class** | **TC #** |
| --- | --- | --- | --- | --- | --- | --- | --- |
| *Ec* | *ycjN* | 2PE | ↑ |  | The ATP-binding Cassette (ABC) Superfamily | ATP-Dependent | 3.A.1 |
| *Ec* | *corC* | 2PE | ↑ |  |  |  |  |
| *Ec* | *sapD* | 2PE | ↑ |  | The ATP-binding Cassette (ABC) Superfamily | ATP-Dependent | 3.A.1 |
| *Ec* | *yjiJ* | 2PE | ↑ | multidrug efflux | The Major Facilitator Superfamily (MFS) | Secondary Transporter | 2.A.1 |
| *Ec* | *yohK* | 2PE | ↑ |  |  |  |  |
| *Ec* | *ygaY* | 2PE | ↑ |  |  |  |  |
| *Ec* | *garP* | 2PE | ↓ | D-glycerate D-glucarate galactarate | The Major Facilitator Superfamily (MFS) | Secondary Transporter | 2.A.1 |
| *Ec* | *yaaJ* | 2PE | ↓ |  | The Alanine or Glycine:Cation Symporter (AGCS) Family | Secondary Transporter | 2.A.25 |
| *Ec* | *ydhP* | 2PE | ↓ |  | The Major Facilitator Superfamily (MFS) | Secondary Transporter | 2.A.1 |
| *Ec* | *yfcJ* | 2PE | ↓ |  | The Major Facilitator Superfamily (MFS) | Secondary Transporter | 2.A.1 |
| *Ec* | *ygdQ* | 2PE | ↓ | tellurium ion efflux | The Tellurium Ion Resistance (TerC) Family | Unclassified | 9.A.30 |
| *Ec* | *yejB* | 2PE | ↓ | ATP ADP phosphate peptide L-alanyl-gamma;-D-glutamyl-meso-diaminopimelate dipeptide | The ATP-binding Cassette (ABC) Superfamily | ATP-Dependent | 3.A.1 |
| *Ec* | *kdpA* | 4Tyr | ↑ | ATP K+ phosphate ADP | The P-type ATPase (P-ATPase) Superfamily | ATP-Dependent | 3.A.3 |
| *Ec* | *sapA* | 4Tyr | ↑ |  | The ATP-binding Cassette (ABC) Superfamily | ATP-Dependent | 3.A.1 |
| *Ec* | *setA* | 4Tyr | ↑ | alpha;-lactose | The Major Facilitator Superfamily (MFS) | Secondary Transporter | 2.A.1 |
| *Ec* | *ydjK* | 4Tyr | ↓ |  | The Major Facilitator Superfamily (MFS) | Secondary Transporter | 2.A.1 |
| *Ec* | *fepD* | 4Tyr | ↓ | ATP ADP phosphate ferric enterobactin complex | The ATP-binding Cassette (ABC) Superfamily | ATP-Dependent | 3.A.1 |
| *Ec* | *wzxE* | 4Tyr | ↓ | N-acetyl-alpha;-D-fucosyl-(1rarr;4)-N-acetyl-beta;-D-mannosaminouronyl-(1rarr;4)-N-acetyl-alpha;-D-glucosaminyl-diphospho-ditrans octacis-undecaprenol | The Multidrug/Oligosaccharidyl-lipid/Polysaccharide (MOP) Flippase Superfamily | Secondary Transporter | 2.A.66 |
| *Ec* | *yjcE* | BenzOH | ↑ |  | The Monovalent Cation:Proton Antiporter-1 (CPA1) Family | Secondary Transporter | 2.A.36 |
| *Ec* | *acrB* | BenzOH | ↑ | drug chenodeoxycholate | The Resistance-Nodulation-Cell Division (RND) Superfamily | Secondary Transporter | 2.A.6 |
| *Ec* | *ygaH* | BenzOH | ↑ | L-valine | The Branched Chain Amino Acid Exporter (LIV-E) Family | Secondary Transporter | 2.A.78 |
| *Ec* | *ycjN* | BenzOH | ↑ |  | The ATP-binding Cassette (ABC) Superfamily | ATP-Dependent | 3.A.1 |
| *Ec* | *kdpA* | BenzOH | ↑ | ATP K+ phosphate ADP | The P-type ATPase (P-ATPase) Superfamily | ATP-Dependent | 3.A.3 |
| *Ec* | *fryC* | BenzOH | ↑ |  | Sugar Specific PTS | Phosphotransferase System (PTS) | 4.A |
| *Ec* | *tolQ* | BenzOH | ↓ |  | The H+- or Na+-translocating Bacterial Flagellar Motor 1ExbBD Outer Membrane Transport Energizer (Mo | Ion Channels | 1.A.30 |
| *Ec* | *manY* | BenzOH | ↓ | HPr - phosphorylated D-mannopyranose D-mannopyranose 6-phosphate | Sugar Specific PTS | Phosphotransferase System (PTS) | 4.A |
| *Ec* | *ybbY* | BenzOH | ↓ |  | The Nucleobase:Cation Symporter-2 (NCS2) Family | Secondary Transporter | 2.A.40 |
| *Ec* | *yjeM* | BenzOH | ↓ |  | The Amino Acid-Polyamine-Organocation (APC) Family | Secondary Transporter | 2.A.3 |
| *Ec* | *yccS* | BenzOH | ↓ | fusaric acid efflux? | The Aromatic Acid Exporter (ArAE) Family | Secondary Transporter | 2.A.85 |
| *Ec* | *yqgA* | Van | ↑ | nitrate nitrite | The Major Facilitator Superfamily (MFS) | Secondary Transporter | 2.A.1 |
| *Ec* | *narU* | Van | ↑ | Na+ (R)-pantothenate | The Solute:Sodium Symporter (SSS) Family | Secondary Transporter | 2.A.21 |
| *Ec* | *panF* | Van | ↑ | fusaric acid efflux? | The Aromatic Acid Exporter (ArAE) Family | Secondary Transporter | 2.A.85 |
| *Ec* | *yeeA* | Van | ↑ | K+ | The Voltage-gated Ion Channel (VIC) Superfamily | Ion Channels | 1.A.1 |
| *Ec* | *kch* | Van | ↓ |  | The ATP-binding Cassette (ABC) Superfamily | ATP-Dependent | 3.A.1 |
| *Ec* | *artI* | Van | ↓ |  | The ATP-binding Cassette (ABC) Superfamily | ATP-Dependent | 3.A.1 |
| *Sc* | *QDR2* | 2PE | ↑ | multidrug efflux? | The Major Facilitator Superfamily (MFS) | Secondary Transporter | 2.A.1 |
| *Sc* | *DRS2* | 2PE | ↑ | Amino phospholipids | The P-type ATPase (P-ATPase) Superfamily | ATP-Dependent | 3.A.3 |
| *Sc* | *ATR1* | 2PE | ↑ | Aminotriazole and 4-nitroquinoline resistance | The Major Facilitator Superfamily (MFS) | Secondary Transporter | 2.A.1 |
| *Sc* | *DNF3* | 2PE | ↑ | calcium ion/phospholipid? | The P-type ATPase (P-ATPase) Superfamily | ATP-Dependent | 3.A.3 |
| *Sc* | *SPF1* | 2PE | ↑ | Involved in sensitivity to Pichia killer toxin | The P-type ATPase (P-ATPase) Superfamily | ATP-Dependent | 3.A.3 |
| *Sc* | *DNF1* | 2PE | ↑ | calcium ion | The P-type ATPase (P-ATPase) Superfamily | ATP-Dependent | 3.A.3 |
| *Sc* | *PMC1* | 2PE | ↑ | Ca2+ (vacuolar) uptake | The P-type ATPase (P-ATPase) Superfamily | ATP-Dependent | 3.A.3 |
| *Sc* | *ATR2* | 2PE | ↑ | aminotriazole? | The Major Facilitator Superfamily (MFS) | Secondary Transporter | 2.A.1 |
| *Sc* | *AGP2* | 2PE | ↓ | Amino acids (general) | The Amino Acid-Polyamine-Organocation (APC) Family | Secondary Transporter | 2.A.3 |
| *Sc* | *FCY2* | 2PE | ↓ | Cytosine/purines | The Nucleobase:Cation Symporter-1 (NCS1) Family | Secondary Transporter | 2.A.39 |
| *Sc* | *CTP1* | 2PE | ↓ | Citrate | The Mitochondrial Carrier (MC) Family | Secondary Transporter | 2.A.29 |
| *Sc* | *YIA6* | 2PE | ↓ | Unclassified | The Mitochondrial Carrier (MC) Family | Secondary Transporter | 2.A.29 |
| *Sc* | *FSF1* | 2PE | ↓ | Tricarboxylates | The Mitochondrial Tricarboxylate Carrier (MTC) Family | Secondary Transporter | 2.A.54 |
| *Sc* | *PTR2* | 2PE | ↓ | Peptides | The Proton-dependent Oligopeptide Transporter (POT) Family | Secondary Transporter | 2.A.17 |
| *Sc* | *YNL095C* | 2PE | ↓ | auxin efflux? | The Auxin Efflux Carrier (AEC) Family | Secondary Transporter | 2.A.69 |
| *Sc* | *MCH4* | 2PE | ↓ | monocarboxylate? | The Major Facilitator Superfamily (MFS) | Secondary Transporter | 2.A.1 |
| *Sc* | *OAC1* | BenzOH | ↑ | oxaloacetate/malonate/sulfate | The Mitochondrial Carrier (MC) Family | Secondary Transporter | 2.A.29 |
| *Sc* | *JEN1* | BenzOH | ↑ | Lactate? | The Major Facilitator Superfamily (MFS) | Secondary Transporter | 2.A.1 |
| *Sc* | *YIA6* | BenzOH | ↑ | Unclassified | The Mitochondrial Carrier (MC) Family | Secondary Transporter | 2.A.29 |
| *Sc* | *PET8* | BenzOH | ↑ | S-adenosylmethionine (SAM) | The Mitochondrial Carrier (MC) Family | Secondary Transporter | 2.A.29 |
| *Sc* | *FLX1* | BenzOH | ↓ | Unclassified | The Mitochondrial Carrier (MC) Family | Secondary Transporter | 2.A.29 |
| *Sc* | *PEX3* | BenzOH | ↓ |  |  |  |  |
| *Sc* | *MCH5* | BenzOH | ↓ | monocarboxylate? | The Major Facilitator Superfamily (MFS) | Secondary Transporter | 2.A.1 |
| *Sc* | *MRX20* | Berb | ↑ | tricarboxylate | The Mitochondrial Carrier (MC) Family | Secondary Transporter | 2.A.29 |
| *Sc* | *AGP2* | Berb | ↑ | Amino acids (general) | The Amino Acid-Polyamine-Organocation (APC) Family | Secondary Transporter | 2.A.3 |
| *Sc* | *FET4* | Berb | ↓ | Fe2+ (uptake; low-affinity) | The Low Affinity Fe2+ Transporter (FeT) Family | Unclassified | 9.A.9 |
| *Sc* | *YOL162W* | Van | ↓ | allantoate? | The Major Facilitator Superfamily (MFS) | Secondary Transporter | 2.A.1 |
| *Sc* | *STV1* | Van | ↓ |  |  |  |  |

**Table S7. List of strains used in this study.** Strains used in the transporters library screening are derived from the parent strains BY4741 (*S. cerevisiae*) and K-12 BW25113 (*E. coli*).

| **Microorganism** | **Strain** | **Genotype** | **Integrative vectors used** | **Reference** |
| --- | --- | --- | --- | --- |
| *Saccharomyces cerevisiae* | CEN.PK113-7D | MATa *URA3 TRP1 LEU2 HIS3* | n.a. | (Entian and Kötter, 2007) |
| *Saccharomyces cerevisiae* | BY4741 | MATa *his3Δ1 leu2Δ0 met15Δ0 ura3Δ0* | n.a. | (Baker Brachmann et al., 1998) |
| *Saccharomyces cerevisiae* | ST9599 | CEN.PK113-7D + *ARO4^K229L^*↑ + *ARO7^G141S^*↑ | pCfB9114 | This study |
| *Saccharomyces cerevisiae* | ST14058 | ST9599 + *FCY2*↑ | pCfB12380 | This study |
| *Saccharomyces cerevisiae* | ST14059 | ST9599 + *MCH4*↑ | pCfB12381 | This study |
| *Saccharomyces cerevisiae* | ST14060 | ST9599 + *PTR2*↑ | pCfB12382 | This study |
| *Saccharomyces cerevisiae* | ST14061 | ST9599 + *YIA6*↑ | pCfB12383 | This study |
| *Saccharomyces cerevisiae* | ST14062 | ST9599 + *YNL095C*↑ | pCfB12384 | This study |
| *Saccharomyces cerevisiae* | ST14063 | ST9599 + *CTP1*↑ | pCfB12385 | This study |
| *Saccharomyces cerevisiae* | ST14064 | ST9599 + *AGP2*↑ | pCfB12386 | This study |
| *Saccharomyces cerevisiae* | ST14065 | ST9599 + *FSF1*↑ | pCfB12387 | This study |
| *Yarrowia lipolytica* | W29, Y-63746 | MATa | n.a. | (Gaillardin et al., 1973) |
| *Escherichia coli* | K-12 MG1655 | *F- lambda- ilvG- rfb-50 rph-1* | n.a. | (Bachmann, 1972) |
| *Escherichia coli* | BL21(DE3) | *B dcm ompT hsdS(rB-mB-) gal* | n.a. | (Studier and Moffatt, 1986) |
| *Escherichia coli* | K-12 BW25113 | *F- DE(araD-araB)567 lacZ4787(del)::rrnB-3 LAM- rph-1 DE(rhaD-rhaB)568 hsdR514* | n.a. | (Baba et al., 2006; Datsenko and Wanner, 2000) |

**Table S8. List of plasmids used in this study**.

| **Plasmid** | **BioBricks** | **Description** | **Reference** |
| --- | --- | --- | --- |
| pCfB3035 | n.a. | Integrative vector for *locus* X-4 | (Jessop‐Fabre et al., 2016) |
| pCfB2909 | n.a. | Integrative vector for *locus* XII-5 | (Jessop‐Fabre et al., 2016) |
| pCfB3042 | n.a. | gRNA vector for *locus* X-4 | (Jessop‐Fabre et al., 2016) |
| pCfB3050 | n.a. | gRNA vector for *locus* XII-5 | (Jessop‐Fabre et al., 2016) |
| pSP-GM1 | n.a. | Plasmid backbone harboring *S. cerevisiae* promoters | (Partow et al., 2010) |
| pCfB9114 | n.a. | Integrative vector for *locus* X-4, expression of *ARO4*K229L and *ARO7*G141S under control of promoter *TEF1* and *PGK1*, respectively | (Babaei et al., 2020) |
| pCfB12380 | BB3034, BB8, BB6709 | Integrative vector for *locus* XII-5, expression of *FCY2* under control of promoter *TEF1* | This study |
| pCfB12381 | BB3034, BB8, BB6710 | Integrative vector for *locus* XII-5, expression of *MCH4* under control of promoter *TEF1* | This study |
| pCfB12382 | BB3034, BB8, BB6711 | Integrative vector for *locus* XII-5, expression of *PTR2* under control of promoter *TEF1* | This study |
| pCfB12383 | BB3034, BB8, BB6712 | Integrative vector for *locus* XII-5, expression of *YIA6* under control of promoter *TEF1* | This study |
| pCfB12384 | BB3034, BB8, BB6713 | Integrative vector for *locus* XII-5, expression of *YNL095C* under control of promoter *TEF1* | This study |
| pCfB12385 | BB3034, BB8, BB6714 | Integrative vector for *locus* XII-5, expression of *CTP1* under control of promoter *TEF1* | This study |
| pCfB12386 | BB3034, BB8, BB6715 | Integrative vector for *locus* XII-5, expression of *AGP2* under control of promoter *TEF1* | This study |
| pCfB12387 | BB3034, BB8, BB6716 | Integrative vector for *locus* XII-5, expression of *FSF1* under control of promoter *TEF1* | This study |

**Table S9. List of BioBricks used in this study**.

| **BioBrick** | **Amplification primer 1** | **Amplification primer 2** | **Template** | **Description** |
| --- | --- | --- | --- | --- |
| BB3034 (XII-5-MarkerFree) | PR-22420 (USER_backbone_1_Rev) | PR-22421 (USER_backbone_2_Fwd) | pCfB2909 | USER-ready backbone of plasmid |
| BB8 (TEF1_U1) | PR-32768 (pTEF1_U1_fwd) | PR-1750 (PTEF1_fw) | pSP-GM1 | *TEF1* promoter for position 1 |
| BB6709 (FCY2_U1) | PR-32769 (FCY2_YER056C_U1_fwd) | PR-32770 (FCY2_YER056C_U1_rv) | CEN.PK113-7D gDNA | *FCY2* gene in position 1 |
| BB6710 (MCH4_U1) | PR-32771 (MCH4_YOL119C_U1_rv) | PR-32772 (MCH4_YOL119C_U1_rv) | CEN.PK113-7D gDNA | *MCH4* gene in position 1 |
| BB6711 (PTR2_U1) | PR-32773 (PTR2_YKR093W_U1_fwd) | PR-32774 (PTR2_YKR093W_U1_rv) | CEN.PK113-7D gDNA | *PTR2* gene in position 1 |
| BB6712 (YIA6_U1) | PR-32775 (YIA6_YIL006W_U1_fwd) | PR-32776 (YIA6_YIL006W_U1_rv) | CEN.PK113-7D gDNA | *YIA6* gene in position 1 |
| BB6713 (YNL095C_U1) | PR-32777 (YNL095C_U1_rv) | PR-32778 (YNL095C_U1_fwd) | CEN.PK113-7D gDNA | *YNL095C* gene in position 1 |
| BB6714 (CTP1_U1) | PR-32779 (CTP1_YBR291C_U1_fwd) | PR-32780 (CTP1_YBR291C_U1_rv) | CEN.PK113-7D gDNA | *CTP1* gene in position 1 |
| BB6715 (AGP2_U1) | PR-32781 (AGP2_YBR132C_U1_fwd) | PR-32782 (AGP2_YBR132C_U1_rv) | CEN.PK113-7D gDNA | *AGP2* gene in position 1 |
| BB6716 (FSF1_U1) | PR-32783 (FSF1_YOR271C_U1_fwd) | PR-32784 (FSF1_YOR271C_U1_rv) | CEN.PK113-7D gDNA | *FSF1* gene in position 1 |

**Table S10. List of primers used in this study**.

| **Primer** | **Sequence** | **Use** |
| --- | --- | --- |
| PR-32768 (pTEF1_U1_fwd) | ACACGCGAUGCACACACCATAGCTTC | Amplification of TEF1 promoter for position 1 |
| PR-1750 (PTEF1_fw) | ACCTGCACUTTGTAATTAAAACTTAGATTAGATTG | Amplification of TEF1 promoter for position 1 |
| PR-22420 (USER_backbone_1_Rev) | ATCGCACGUGTAGATACGTTGTTGACACTTC | Amplification of EasyClone integrative vectors |
| PR-22421 (USER_backbone_2_Fwd) | ATCGCGTGUATCCGCTCTAACCGAAAAGGAAG | Amplification of EasyClone integrative vectors |
| PR-32769 (FCY2_YER056C_U1_fwd) | AGTGCAGGUAAAACAATGTTGGAAGAGGGAAATAATGTTTACG | Amplification of FCY2 promoter for position 1 |
| PR-32770 (FCY2_YER056C_U1_rv) | ACGTGCGAUCTAACGACCGAAGTATTTCAATTCTAAAGG | Amplification of FCY2 promoter for position 1 |
| PR-32771 (MCH4_YOL119C_U1_rv) | AGTGCAGGUAAAACAATGTTGAACATTCCCATAATTGCTAACTCC | Amplification of MCH4 promoter for position 1 |
| PR-32772 (MCH4_YOL119C_U1_rv) | ACGTGCGAUTTAAAACTTACAAAGCTTCGCACCAAC | Amplification of MCH4 promoter for position 1 |
| PR-32773 (PTR2_YKR093W_U1_fwd) | AGTGCAGGUAAAACAATGCTCAACCATCCCAGCC | Amplification of PTR2 promoter for position 1 |
| PR-32774 (PTR2_YKR093W_U1_rv) | ACGTGCGAUCTAATATTTGGTGGTGGATCTTAGACTTTCC | Amplification of PTR2 promoter for position 1 |
| PR-32775 (YIA6_YIL006W_U1_fwd) | AGTGCAGGUAAAACAATGACACAGACTGATAATCCTGTCCC | Amplification of YIA6 promoter for position 1 |
| PR-32776 (YIA6_YIL006W_U1_rv) | ACGTGCGAUTTAAATTACCATAGTGCTAATATTTTCTAGGCGG | Amplification of YIA6 promoter for position 1 |
| PR-32777 (YNL095C_U1_rv) | AGTGCAGGUAAAACAATGGTGCACATTACTCTGGGTC | Amplification of YNL095C promoter for position 1 |
| PR-32778 (YNL095C_U1_fwd) | ACGTGCGAUTAAAGGTTCATCTGTACTTTCAGAAAGTAAG | Amplification of YNL095C promoter for position 1 |
| PR-32779 (CTP1_YBR291C_U1_fwd) | ACGTGCGAUTCAGGCTAGCATAACTAAGACCTTTTCATAG | Amplification of CTP1 promoter for position 1 |
| PR-32780 (CTP1_YBR291C_U1_rv) | AGTGCAGGUAAAACAATGTCCAGTAAAGCTACCAAAAGTGAC | Amplification of CTP1 promoter for position 1 |
| PR-32781 (AGP2_YBR132C_U1_fwd) | AGTGCAGGUAAAACAATGACAAAGGAACGTATGACCATCG | Amplification of AGP2 promoter for position 1 |
| PR-32782 (AGP2_YBR132C_U1_rv) | ACGTGCGAUTTATGCTTTGCTATAATATTGAAATTTTTCGAAGG | Amplification of AGP2 promoter for position 1 |
| PR-32783 (FSF1_YOR271C_U1_fwd) | AGTGCAGGUAAAACAATGGCATCATCAGTCCCAGG | Amplification of FSF1 promoter for position 1 |
| PR-32784 (FSF1_YOR271C_U1_rv) | ACGTGCGAUCTAAATACCTCTGTTAAAATAGACCTTTTCAATTGG | Amplification of FSF1 promoter for position 1 |

**Figure S1. Toxicity of 54 aromatic compounds evaluated in *S. cerevisiae*, *Y. lipolytica* and *E. coli***. µ_max_ was determined as the highest slope of ln-transformed OD_600_ versus time within the exponential growth phase. The µ_max_ covered a window of 12 time points, and required a minimum R^2^ value of 0.98. Error bars represent the standard deviation from two biological replicates. *: Addition of the given compound causes medium blackening and impedes µ_max_ estimation.

**Figure S2. Box and Whisker diagram of transporters deletion library screening.** Strains unable to grow in the control medium were excluded from the analysis, resulting in the selection of 444 strains in *E. coli* and 305 in *S. cerevisiae*. µ_max_ is calculated as the mean of two biological replicates.

**Figure S3. Validated transporter deletions that either improve or reduce the tolerance of a) *E. coli* and b) *S. cerevisiae* to multiple toxic aromatic compounds.** *Control* represents µ_max_ with respect to the wild-type strain in control medium, lacking any toxic aromatic compound. *Aromatic compound* shows the µ_max_ with respect to the wild-type strain in the same medium but containing the aromatic compound indicated in each subplot, at the same concentration used in the library screening (Table S9). Error bars denote the estimated standard deviation of at least 4 biological replicates. The horizontal dashed line indicates a value of 1, expected for strains not showing differences compared to the wild-type control.

**Figure S4. Growth profiles of validated transporter deletions improving or reducing tolerance.** A representative growth curve per strain is shown. Font colors of deleted transporter-encoding genes represent the compound. a) *E. coli*. Blue: 2-phenylethanol; orange: 4-tyrosol; green: benzyl alcohol; red: vanillin. b) *S. cerevisiae.* Blue: 2-phenylethanol; orange: benzyl alcohol; green: berberine; red: vanillin.

**Figure S5. Clustermap showing highly correlated transporter deletions across four different aromatic compounds in (a) *E. coli* and (b) *S. cerevisiae*.** The clustering parameter is the mean of relative and normalized µmax, which compares the µmax in the presence of the toxic aromatic compound to the wild-type control and normalizes it against the effect of the deletion in the absence of the compound. The heatmap values are scaled from 0 to 1 for better comparability and interpretation. For each transporter pair, the correlation scatterplots are presented in Figures S6-S7.

**Figure S6. Pairs of transporters-encoding genes showing high degree of correlation in *E. coli*.** The mean relative and normalized µ_max_ is used as correlation parameter. It compares the µ_max_ in the presence of the toxic aromatic compound to the wild-type control and normalizes it against the effect of the deletion in the absence of the compound. A R^2^ coefficient higher than 0.999 and a p-value < 0.05 after a Bonferroni correction was enforced for the selection of the pairs. Colors indicate the different compounds: Blue: 2-phenylethanol; orange: 4-tyrosol; green: benzyl alcohol; red: vanillin.

**Figure S7. Pairs of transporters-encoding genes showing high degree of correlation in *S. cerevisiae*.** The mean relative and normalized µ_max_ is used as correlation parameter. It compares the µ_max_ in the presence of the toxic aromatic compound to the wild-type control and normalizes it against the effect of the deletion in the absence of the compound. A R^2^ coefficient higher than 0.999 and a p-value < 0.05 after a Bonferroni correction was enforced for the selection of the pairs. Colors indicate the different compounds: Blue: 2-phenylethanol; orange: benzyl alcohol; green: berberine; red: vanillin.

**Figure S8. Growth profiles of transporter deletions improving or reducing 2-phenylethanol tolerance in an L-phenylalanine bioconversion process.** A representative growth curve per strain is shown. L-phenylalanine was supplemented at a concentration of 5 g/L. Top panel: transporter deletions improving 2-phenylethanol tolerance. Middle panel: transporter deletions reducing 2-phenylethanol tolerance. Bottom panel: overexpression of transporters that upon deletion reduce 2-phenylethanol tolerance.

**References**

Aguilar-Zárate, P., Cruz, M.A., Montañez, J., Rodríguez-Herrera, R., Wong-Paz, J.E., Belmares, R.E., Aguilar, C.N., 2015. Gallic acid production under anaerobic submerged fermentation by two bacilli strains. Microb. Cell Factories 14, 209. https://doi.org/10.1186/s12934-015-0386-2

Ahmadi, M.K., Fang, L., Moscatello, N., Pfeifer, B.A., 2016. E. coli metabolic engineering for gram scale production of a plant-based anti-inflammatory agent. Metab. Eng. 38, 382–388. https://doi.org/10.1016/j.ymben.2016.10.001

Averesch, N.J.H., Winter, G., Krömer, J.O., 2016. Production of para-aminobenzoic acid from different carbon-sources in engineered Saccharomyces cerevisiae. Microb. Cell Factories 15, 89. https://doi.org/10.1186/s12934-016-0485-8

Baba, T., Ara, T., Hasegawa, M., Takai, Y., Okumura, Y., Baba, M., Datsenko, K.A., Tomita, M., Wanner, B.L., Mori, H., 2006. Construction of *Escherichia coli* K‐12 in‐frame, single‐gene knockout mutants: the Keio collection. Mol. Syst. Biol. 2. https://doi.org/10.1038/msb4100050

Babaei, M., Borja Zamfir, G.M., Chen, X., Christensen, H.B., Kristensen, M., Nielsen, J., Borodina, I., 2020. Metabolic Engineering of *Saccharomyces cerevisiae* for Rosmarinic Acid Production. ACS Synth. Biol. 9, 1978–1988. https://doi.org/10.1021/acssynbio.0c00048

Bachmann, B.J., 1972. Pedigrees of some mutant strains of Escherichia coli K-12. Bacteriol. Rev. 36, 525–557. https://doi.org/10.1128/br.36.4.525-557.1972

Baker Brachmann, C., Davies, A., Cost, G.J., Caputo, E., Li, J., Hieter, P., Boeke, J.D., 1998. Designer deletion strains derived fromSaccharomyces cerevisiae S288C: A useful set of strains and plasmids for PCR-mediated gene disruption and other applications. Yeast 14, 115–132. https://doi.org/10.1002/(SICI)1097-0061(19980130)14:2<115::AID-YEA204>3.0.CO;2-2

Brochado, A.R., Matos, C., Møller, B.L., Hansen, J., Mortensen, U.H., Patil, K.R., 2010. Improved vanillin production in baker’s yeast through in silico design. Microb. Cell Factories 9, 84. https://doi.org/10.1186/1475-2859-9-84

Datsenko, K.A., Wanner, B.L., 2000. One-step inactivation of chromosomal genes in *Escherichia coli* K-12 using PCR products. Proc. Natl. Acad. Sci. 97, 6640–6645. https://doi.org/10.1073/pnas.120163297

De Bruyn, F., Van Brempt, M., Maertens, J., Van Bellegem, W., Duchi, D., De Mey, M., 2015. Metabolic engineering of Escherichia coli into a versatile glycosylation platform: production of bio-active quercetin glycosides. Microb. Cell Factories 14, 138. https://doi.org/10.1186/s12934-015-0326-1

Elbourne, L.D.H., Tetu, S.G., Hassan, K.A., Paulsen, I.T., 2017. TransportDB 2.0: a database for exploring membrane transporters in sequenced genomes from all domains of life. Nucleic Acids Res. 45, D320–D324. https://doi.org/10.1093/nar/gkw1068

Entian, K.-D., Kötter, P., 2007. 25 Yeast Genetic Strain and Plasmid Collections, in: Stansfield, I., Stark, M.J. (Eds.), Methods in Microbiology, Yeast Gene Analysis. Academic Press, pp. 629–666. https://doi.org/10.1016/S0580-9517(06)36025-4

Falkenberg, K.B., Mol, V., de la Maza Larrea, A.S., Pogrebnyakov, I., Nørholm, M.H.H., Nielsen, A.T., Jensen, S.I., 2021. The ProUSER2.0 Toolbox: Genetic Parts and Highly Customizable Plasmids for Synthetic Biology in *Bacillus subtilis*. ACS Synth. Biol. 10, 3278–3289. https://doi.org/10.1021/acssynbio.1c00130

Fordjour, E., Adipah, F.K., Zhou, S., Du, G., Zhou, J., 2019. Metabolic engineering of Escherichia coli BL21 (DE3) for de novo production of l-DOPA from d-glucose. Microb. Cell Factories 18, 74. https://doi.org/10.1186/s12934-019-1122-0

Gaillardin, C.M., Charoy, V., Heslot, H., 1973. A study of copulation, sporulation and meiotic segregation in Candida lipolytica. Arch. Für Mikrobiol. 92, 69–83. https://doi.org/10.1007/BF00409513

Giaever, G., Chu, A.M., Ni, L., Connelly, C., Riles, L., Véronneau, S., Dow, S., Lucau-Danila, A., Anderson, K., André, B., Arkin, A.P., Astromoff, A., El Bakkoury, M., Bangham, R., Benito, R., Brachat, S., Campanaro, S., Curtiss, M., Davis, K., Deutschbauer, A., Entian, K.-D., Flaherty, P., Foury, F., Garfinkel, D.J., Gerstein, M., Gotte, D., Güldener, U., Hegemann, J.H., Hempel, S., Herman, Z., Jaramillo, D.F., Kelly, D.E., Kelly, S.L., Kötter, P., LaBonte, D., Lamb, D.C., Lan, N., Liang, H., Liao, H., Liu, L., Luo, C., Lussier, M., Mao, R., Menard, P., Ooi, S.L., Revuelta, J.L., Roberts, C.J., Rose, M., Ross-Macdonald, P., Scherens, B., Schimmack, G., Shafer, B., Shoemaker, D.D., Sookhai-Mahadeo, S., Storms, R.K., Strathern, J.N., Valle, G., Voet, M., Volckaert, G., Wang, C., Ward, T.R., Wilhelmy, J., Winzeler, E.A., Yang, Y., Yen, G., Youngman, E., Yu, K., Bussey, H., Boeke, J.D., Snyder, M., Philippsen, P., Davis, R.W., Johnston, M., 2002. Functional profiling of the Saccharomyces cerevisiae genome. Nature 418, 387–391. https://doi.org/10.1038/nature00935

Gietz, R.D., Schiestl, R.H., 2007. High-efficiency yeast transformation using the LiAc/SS carrier DNA/PEG method. Nat. Protoc. 2, 31–34. https://doi.org/10.1038/nprot.2007.13

Gold, N.D., Gowen, C.M., Lussier, F.-X., Cautha, S.C., Mahadevan, R., Martin, V.J.J., 2015. Metabolic engineering of a tyrosine-overproducing yeast platform using targeted metabolomics. Microb. Cell Factories 14, 73. https://doi.org/10.1186/s12934-015-0252-2

Hamana, K., Niitsu, M., 1999. Production of 2-phenylethylamine by decarboxylation of L-phenylalanine in alkaliphilic Bacillus cohnii. J. Gen. Appl. Microbiol. 45, 149–153. https://doi.org/10.2323/jgam.45.149

Han, J., Li, S., 2023. De novo biosynthesis of berberine and halogenated benzylisoquinoline alkaloids in Saccharomyces cerevisiae. Commun. Chem. 6, 27. https://doi.org/10.1038/s42004-023-00821-9

Jamil, O.K., Cravens, A., Payne, J.T., Kim, C.Y., Smolke, C.D., 2022. Biosynthesis of tetrahydropapaverine and semisynthesis of papaverine in yeast. Proc. Natl. Acad. Sci. 119, e2205848119. https://doi.org/10.1073/pnas.2205848119

Jensen, N.B., Strucko, T., Kildegaard, K.R., David, F., Maury, J., Mortensen, U.H., Forster, J., Nielsen, J., Borodina, I., 2014. EasyClone: method for iterative chromosomal integration of multiple genes Saccharomyces cerevisiae. FEMS Yeast Res. 14, 238–248. https://doi.org/10.1111/1567-1364.12118

Jessop‐Fabre, M.M., Jakočiūnas, T., Stovicek, V., Dai, Z., Jensen, M.K., Keasling, J.D., Borodina, I., 2016. EasyClone-MarkerFree: A vector toolkit for marker-less integration of genes into Saccharomyces cerevisiae via CRISPR-Cas9. Biotechnol. J. 11, 1110–1117. https://doi.org/10.1002/biot.201600147

Lee, K., Bang, H.B., Lee, Y.H., Jeong, K.J., 2019. Enhanced production of styrene by engineered Escherichia coli and in situ product recovery (ISPR) with an organic solvent. Microb. Cell Factories 18, 79. https://doi.org/10.1186/s12934-019-1129-6

Leonard, E., Yan, Y., Koffas, M., 2006. Functional expression of a P450 flavonoid hydroxylase for the biosynthesis of plant-specific hydroxylated flavonols in Escherichia coli. Metab. Eng. 8, 172–181. https://doi.org/10.1016/j.ymben.2005.11.001

Li, J., Ye, B.-C., 2021. Metabolic engineering of Pseudomonas putida KT2440 for high-yield production of protocatechuic acid. Bioresour. Technol. 319, 124239. https://doi.org/10.1016/j.biortech.2020.124239

Liu, M., Wang, C., Ren, X., Gao, S., Yu, S., Zhou, J., 2022. Remodelling metabolism for high-level resveratrol production in Yarrowia lipolytica. Bioresour. Technol. 365, 128178. https://doi.org/10.1016/j.biortech.2022.128178

Liu, X., Liu, J., Lei, D., Zhao, G.-R., 2022. Modular metabolic engineering for production of phloretic acid, phloretin and phlorizin in Escherichia coli. Chem. Eng. Sci. 247, 116931. https://doi.org/10.1016/j.ces.2021.116931

Liu, X., Niu, H., Li, Q., Gu, P., 2019. Metabolic engineering for the production of l-phenylalanine in Escherichia coli. 3 Biotech 9, 85. https://doi.org/10.1007/s13205-019-1619-6

Liu, Z., Zhang, X., Lei, D., Qiao, B., Zhao, G.-R., 2021. Metabolic engineering of Escherichia coli for de novo production of 3-phenylpropanol via retrobiosynthesis approach. Microb. Cell Factories 20, 121. https://doi.org/10.1186/s12934-021-01615-1

Lv, H., Zhang, Y., Shao, J., Liu, H., Wang, Y., 2021. Ferulic acid production by metabolically engineered Escherichia coli. Bioresour. Bioprocess. 8, 70. https://doi.org/10.1186/s40643-021-00423-0

Lyu, X., Zhao, G., Ng, K.R., Mark, R., Chen, W.N., 2019. Metabolic Engineering of *Saccharomyces cerevisiae* for De Novo Production of Kaempferol. J. Agric. Food Chem. 67, 5596–5606. https://doi.org/10.1021/acs.jafc.9b01329

Martínez, J.A., Bolívar, F., Escalante, A., 2015. Shikimic Acid Production in Escherichia coli: From Classical Metabolic Engineering Strategies to Omics Applied to Improve Its Production. Front. Bioeng. Biotechnol. 3. https://doi.org/10.3389/fbioe.2015.00145

Mora-Villalobos, J.-A., Zeng, A.-P., 2018. Synthetic pathways and processes for effective production of 5-hydroxytryptophan and serotonin from glucose in Escherichia coli. J. Biol. Eng. 12, 3. https://doi.org/10.1186/s13036-018-0094-7

Munro, L.J., Kell, D.B., 2022. Analysis of a Library of Escherichia coli Transporter Knockout Strains to Identify Transport Pathways of Antibiotics. Antibiotics 11, 1129. https://doi.org/10.3390/antibiotics11081129

Niu, H., Li, R., Liang, Q., Qi, Q., Li, Q., Gu, P., 2019. Metabolic engineering for improving L-tryptophan production in *Escherichia coli*. J. Ind. Microbiol. Biotechnol. 46, 55–65. https://doi.org/10.1007/s10295-018-2106-5

Partow, S., Siewers, V., Bjørn, S., Nielsen, J., Maury, J., 2010. Characterization of different promoters for designing a new expression vector in Saccharomyces cerevisiae. Yeast 27, 955–964. https://doi.org/10.1002/yea.1806

Pugh, S., McKenna, R., Halloum, I., Nielsen, D.R., 2015. Engineering Escherichia coli for renewable benzyl alcohol production. Metab. Eng. Commun. 2, 39–45. https://doi.org/10.1016/j.meteno.2015.06.002

Rodriguez, A., Kildegaard, K.R., Li, M., Borodina, I., Nielsen, J., 2015. Establishment of a yeast platform strain for production of p-coumaric acid through metabolic engineering of aromatic amino acid biosynthesis. Metab. Eng. 31, 181–188. https://doi.org/10.1016/j.ymben.2015.08.003

Rodriguez, A., Strucko, T., Stahlhut, S.G., Kristensen, M., Svenssen, D.K., Forster, J., Nielsen, J., Borodina, I., 2017. Metabolic engineering of yeast for fermentative production of flavonoids. Bioresour. Technol. 245, 1645–1654. https://doi.org/10.1016/j.biortech.2017.06.043

Salgado, J.M., Rodríguez-Solana, R., Curiel, J.A., de las Rivas, B., Muñoz, R., Domínguez, J.M., 2014. Bioproduction of 4-vinylphenol from corn cob alkaline hydrolyzate in two-phase extractive fermentation using free or immobilized recombinant E. coli expressing pad gene. Enzyme Microb. Technol. 58–59, 22–28. https://doi.org/10.1016/j.enzmictec.2014.02.005

Song, G., Wu, F., Peng, Y., Jiang, X., Wang, Q., 2022. High-Level Production of Catechol from Glucose by Engineered Escherichia coli. Fermentation 8, 344. https://doi.org/10.3390/fermentation8070344

Steffensky, M., Mühlenweg, A., Wang, Z.-X., Li, S.-M., Heide, L., 2000. Identification of the Novobiocin Biosynthetic Gene Cluster of *Streptomyces spheroides* NCIB 11891. Antimicrob. Agents Chemother. 44, 1214–1222. https://doi.org/10.1128/AAC.44.5.1214-1222.2000

Studier, F.W., Moffatt, B.A., 1986. Use of bacteriophage T7 RNA polymerase to direct selective high-level expression of cloned genes. J. Mol. Biol. 189, 113–130. https://doi.org/10.1016/0022-2836(86)90385-2

Sun, Z., Ning, Y., Liu, L., Liu, Y., Sun, B., Jiang, W., Yang, C., Yang, S., 2011. Metabolic engineering of the L-phenylalanine pathway in Escherichia coli for the production of S- or R-mandelic acid. Microb. Cell Factories 10, 71. https://doi.org/10.1186/1475-2859-10-71

Trenchard, I.J., Siddiqui, M.S., Thodey, K., Smolke, C.D., 2015. De novo production of the key branch point benzylisoquinoline alkaloid reticuline in yeast. Metab. Eng. 31, 74–83. https://doi.org/10.1016/j.ymben.2015.06.010

Vargas-Tah, A., Gosset, G., 2015. Production of Cinnamic and p-Hydroxycinnamic Acids in Engineered Microbes. Front. Bioeng. Biotechnol. 3. https://doi.org/10.3389/fbioe.2015.00116

Wang, G., Tavares, A., Schmitz, S., França, L., Almeida, H., Cavalheiro, J., Carolas, A., Øzmerih, S., Blank, L.M., Ferreira, B.S., Borodina, I., 2022. An integrated yeast‐based process for *cis* , *cis* ‐muconic acid production. Biotechnol. Bioeng. 119, 376–387. https://doi.org/10.1002/bit.27992

Wang, G., Wang, M., Yang, J., Li, Q., Zhu, N., Liu, L., Hu, X., Yang, X., 2023. *De novo* Synthesis of 2-phenylethanol from Glucose by Metabolically Engineered *Escherichia coli*. J. Ind. Microbiol. Biotechnol. 49, kuac026. https://doi.org/10.1093/jimb/kuac026

Wang, H., Liu, W., Shi, F., Huang, L., Lian, J., Qu, L., Cai, J., Xu, Z., 2018. Metabolic pathway engineering for high-level production of 5-hydroxytryptophan in Escherichia coli. Metab. Eng. 48, 279–287. https://doi.org/10.1016/j.ymben.2018.06.007

Wang, Q., Wu, X., Lu, X., He, Y., Ma, B., Xu, Y., 2021. Efficient Biosynthesis of Vanillin from Isoeugenol by Recombinant Isoeugenol Monooxygenase from Pseudomonas nitroreducens Jin1. Appl. Biochem. Biotechnol. 193, 1116–1128. https://doi.org/10.1007/s12010-020-03478-5

Wang, Y., Zhang, H., Lu, X., Zong, H., Zhuge, B., 2019. Advances in 2-phenylethanol production from engineered microorganisms. Biotechnol. Adv. 37, 403–409. https://doi.org/10.1016/j.biotechadv.2019.02.005

Wei, W., Zhang, P., Shang, Y., Zhou, Y., Ye, B.-C., 2020. Metabolically engineering of Yarrowia lipolytica for the biosynthesis of naringenin from a mixture of glucose and xylose. Bioresour. Technol. 314, 123726. https://doi.org/10.1016/j.biortech.2020.123726

Weiland, F., Barton, N., Kohlstedt, M., Becker, J., Wittmann, C., 2023. Systems metabolic engineering upgrades Corynebacterium glutamicum to high-efficiency cis, cis-muconic acid production from lignin-based aromatics. Metab. Eng. 75, 153–169. https://doi.org/10.1016/j.ymben.2022.12.005

Winzeler, E.A., Shoemaker, D.D., Astromoff, A., Liang, H., Anderson, K., Andre, B., Bangham, R., Benito, R., Boeke, J.D., Bussey, H., Chu, A.M., Connelly, C., Davis, K., Dietrich, F., Dow, S.W., El Bakkoury, M., Foury, F., Friend, S.H., Gentalen, E., Giaever, G., Hegemann, J.H., Jones, T., Laub, M., Liao, H., Liebundguth, N., Lockhart, D.J., Lucau-Danila, A., Lussier, M., M’Rabet, N., Menard, P., Mittmann, M., Pai, C., Rebischung, C., Revuelta, J.L., Riles, L., Roberts, C.J., Ross-MacDonald, P., Scherens, B., Snyder, M., Sookhai-Mahadeo, S., Storms, R.K., Véronneau, S., Voet, M., Volckaert, G., Ward, T.R., Wysocki, R., Yen, G.S., Yu, K., Zimmermann, K., Philippsen, P., Johnston, M., Davis, R.W., 1999. Functional Characterization of the *S. cerevisiae* Genome by Gene Deletion and Parallel Analysis. Science 285, 901–906. https://doi.org/10.1126/science.285.5429.901

Xu, W., Yang, C., Xia, Y., Zhang, L., Liu, C., Yang, H., Shen, W., Chen, X., 2020. High-Level Production of Tyrosol with Noninduced Recombinant *Escherichia coli* by Metabolic Engineering. J. Agric. Food Chem. 68, 4616–4623. https://doi.org/10.1021/acs.jafc.9b07610

Yin, H., Hu, T., Zhuang, Y., Liu, T., 2020. Metabolic engineering of Saccharomyces cerevisiae for high-level production of gastrodin from glucose. Microb. Cell Factories 19, 218. https://doi.org/10.1186/s12934-020-01476-0

Zhang, Y., He, Y., Zhang, N., Gan, J., Zhang, S., Dong, Z., 2021. Combining protein and metabolic engineering strategies for biosynthesis of melatonin in Escherichia coli. Microb. Cell Factories 20, 170. https://doi.org/10.1186/s12934-021-01662-8

Zhao, S., Jones, J.A., Lachance, D.M., Bhan, N., Khalidi, O., Venkataraman, S., Wang, Z., Koffas, M.A.G., 2015. Improvement of catechin production in Escherichia coli through combinatorial metabolic engineering. Metab. Eng. 28, 43–53. https://doi.org/10.1016/j.ymben.2014.12.002

Zhou, P., Yue, C., Shen, B., Du, Y., Xu, N., Ye, L., 2021. Metabolic engineering of Saccharomyces cerevisiae for enhanced production of caffeic acid. Appl. Microbiol. Biotechnol. 105, 5809–5819. https://doi.org/10.1007/s00253-021-11445-1
